# Microbial communities associated with plastic fishing nets: diversity, potentially pathogenic and hydrocarbon degrading bacteria

**DOI:** 10.1038/s41598-025-06033-6

**Published:** 2025-07-02

**Authors:** Rafaela Perdigão, Ana Sofia Tavares, Maria F. Carvalho, Catarina Magalhães, Sandra Ramos, C. Marisa R. Almeida, Ana P. Mucha

**Affiliations:** 1 CIIMAR/CIMAR LA, Interdisciplinary Centre of Marine and Environmental Research, University of Porto. Terminal de Cruzeiros do Porto de Leixões, Avenida General Norton de Matos, S/N, Matosinhos, 4450-208 Portugal; 2 ICBAS - School of Medicine and Biomedical Sciences, University of Porto, Rua de Jorge Viterbo Ferreira, 228, Porto, 4050-313 Portugal; 3 Faculty of Sciences, University of Porto, Rua do Campo Alegre 790, Porto, 4150-171 Portugal

**Keywords:** Fishing Nets, Microbial communities, Plastic, Polyethylene, Nylon, Pathogens, Hydrocarbon degraders, Maritime Port, Marine microbiology, Environmental biotechnology, Microbial communities

## Abstract

Abandoned, lost or otherwise discarded fishing gear (ALDFG) represent a major source of marine plastic litter pollution. Similar to other plastic litter, these items can provide a new surface for the growth of biofilms harboring distinct microbial communities, containing potential opportunistic pathogens or pollutant-degrading microorganisms. While knowledge is increasing for marine plastic litter and microplastic-associated biofilms, there is a gap on the plastisphere research for fishing gear. This study aimed to comprehend the structure and dynamics of the microbial communities attached to plastic fishing nets, mimicking a scenario when lost at sea, but also to assess if polymer type can influence these communities. For that, a one-year in situ experiment was employed inside a recreational marina (port of Leixões, Portugal), using 3 types of plastic fishing nets (Braided Polyethylene (PE), Braided Nylon and Thin Nylon) submersed in the seawater. Seasonal samplings of nets and surrounding seawater were performed for microbial community analysis by 16 S rRNA metabarcoding. One month-old-nets samples were additionally collected for cultivation of bacterial strains in the laboratory. In general, microbial communities found in the biofilms attached to fishing nets were taxonomically distinct and more diverse, when compared to the surrounding seawater. Biofilm communities were not shaped by the polymer type, instead, they displayed a succession pattern over time. Biofilm communities were predominantly composed of the phyla Proteobacteria, Bacteroidetes and Verrucomicrobiota. Additionally, the families *Sphingomonadaceae*, *Rubritaleaceae*, *Rhizobiaceae* and *Saprospiraceae* were specifically associated with fishing net biofilms. From the 3 nets, a total of 123 bacterial strains from 46 bacterial genera were recovered. The genera *Acinetobacter*, *Bacillus*, *Rhodococcus*, *Shewanella*, *Streptomyces* and *Vibrio* were common to all nets. Commonly associated hydrocarbon and plastic - degrading taxa were highly abundant in the biofilm communities (> 2% abundance) and some were even possible to cultivate in laboratory. In addition, biofilm communities presented as well, potentially pathogenic genera, such as *Clostridium* and *Mycobacterium*, but in low abundances (< 1%). With this work, a deeper knowledge on the plastisphere associated with different plastic fishing gear was obtained, along with the isolation of bacterial strains with potential for future exploration of plastic biodegradation.

**Supplementary Information:**

The online version contains supplementary material available at 10.1038/s41598-025-06033-6.

## Introduction

Abandoned, lost, or otherwise discarded fishing gear (ALDFG), contributes heavily to the marine litter problematic in our ocean. Although it is extremely difficult, to quantify the amount of ALDFG entering the ocean^1^, Kuczenski et al.^2^ estimated a global loss of about 48.4 kt of fishing gear, for industrial fisheries alone (trawl, purse-seine and pelagic longline fisheries), in 2018. By collecting trawl samples across the Great Pacific Garbage Patch (GPGP), Lebreton et al.^3^ observed that fishing nets represented at least 46% of the floating debris in that area.

Regardless of the numbers, ALDFG pose undeniable threats to marine ecosystems, since they can accumulate other litter items, cause entanglement of marine wildlife and carry invasive species^4–7^. Being mostly made of plastic, ALDFG may also adsorb contaminants such as metals and organic pollutants^8–11^, be a potential transportation vector for pathogenic agents^12,13^, and release microplastics to the environment^14–16^. Given the high durability of plastic materials, lost fishing gear can continue fishing for a long time (ghost fishing) depending on the area and conditions they were lost in^17,18^.

By offering a new surface for microorganisms to attach, plastic marine debris presents also a unique world of organisms, referred to as the plastisphere by Zettler et al.^19^. These communities are distinct from the surrounding seawater ones and while some authors report that polymer types dictate the communities’ structure^20–22^, others suggest that location, environmental parameters, and weathering conditions of the plastic are important factors driving the community changes in plastic biofilms^23,24^. Members of the phyla Proteobacteria (*Rhodobacteraceae*,* Sphingomonadaceae*, *Hyphomonadaceae* and *Alteromonadaceae* families) Bacteroidota (*Flavobacteriaceae* and *Cyclobacteriaceae* families), Firmicutes (or Bacillota), and Cyanobacteria are repeatedly pointed out as core members of the plastisphere^19,25–28^. When addressing the pathogenicity of plastic-associated communities, members of *Vibrio*,* Escherichia* and *Arcobacter* are frequently reported^13,29,30^ and the question to whether plastic or microplastic-associated communities represent a vector also of antimicrobial resistance genes, is of increasing concern^12,31^.

From the study of the plastisphere, researchers do not want solely to unravel the microbial community’s succession or determine their potential to carry pathogens, also, taxa with plastic-degradation potential are a topic of interest^32,33^.

The presence of well-known hydrocarbon degrading microorganisms in plastic biofilms^20,22,24,34^, has raised interest and the question to whether these taxa might have also the potential to degrade fossil-based plastics, or other pollutants there attached. In fact, xenobiotic degradation pathways, namely for aromatic hydrocarbons, have been reported to be enriched on the polymer-associated communities^28,35^. Studies have demonstrated so far, plastic-degradation potential for species of the genera *Alcanivorax*, *Bacillus*, *Erythrobacter*, *Exiguobacterium*, *Pseudomonas*, and *Streptomyces*^36–42^.

Most studies on plastic-associated communities focus on plastic debris and/or microplastics collected from several locations, either open sea, coastal areas or even the deep-sea. While prior studies have explored biofilm communities on fishing gear^43,44^, none have systematically compared different gear types (polymers, twine thickness) to assess their environmental impacts. To fill that gap we aimed to evaluate in situ the succession of microbial communities associated with plastic fishing nets, in seawater, with special emphasis on (i) the identification of hydrocarbon- and plastic-degrading bacteria, and (ii) the presence/accumulation of harmful microorganisms such as opportunistic pathogens.

## Materials and methods

### In situ experimental set up and sampling

An in situ experiment was carried out, to study the structure and dynamics of microbial communities associated with plastic fishing nets, and their potential to harbor pollutant-degrading bacteria and carry pathogens, once lost at sea. For this study, 3 new fishing nets, supplied by a manufacturer of fishing gear, were exposed to quasi-real environmental conditions, inside the recreational marina of Leixões (Matosinhos, Portugal). The characterization of each net is described in Table 1.

**Table 1:** Characterization of plastic fishing Nets used in the in situ experiment at marina of leixões, matosinhos.

	Net A – Braided PE	Net B - Braided Nylon	Net C - Thin Nylon
		
Main use in fishing	Trawling net	Seine net	Seine net
Material	High Density Polyethylene fibres	Nylon 6,6 fibres	Nylon 6,6 fibres
Color	Green	White	Grey
Rope assembly	Braided	Braided	Monofilament
Diameter (mm)	3	2.3	0.3
Mesh size (mm)	150	53	100

**Fig. 1:**
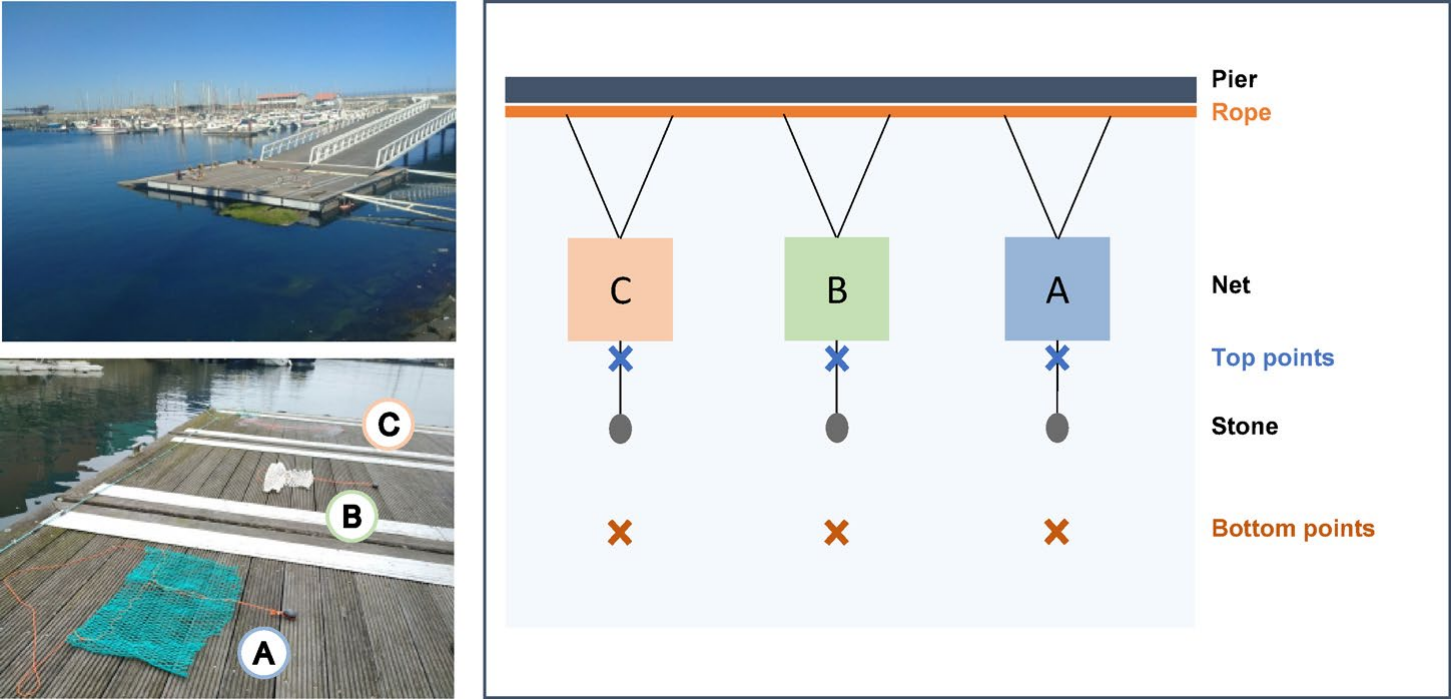
Experimental design of the in situ experiment at marina of Leixões, Matosinhos, with the Braided PE (Net A), Braided Nylon (Net B) and Thin Nylon (Net C) nets.

The three nets, namely Braided PE (Net A), Braided Nylon (Net B) and Thin Nylon (Net C), were distanced 50 cm apart from each other. To guarantee a complete submersion of the fishing nets, at approximately 50 cm below the surface, each net was kept transversally open with a rope entangled on the top, which was connected to a stone at the bottom. The top ropes were then linked to another of about 9 m, that stayed attached onto the wooden pier of the marina. The overall experimental design is represented in (Fig. 1).

The in situ experiment began in February 2020, and lasted for a year with the collection of seawater samples at the beginning (NL0220), and samples of both nets and seawater in the months of March (NL0320), May (NL0520), July (NL0720), October 2020 (NL1020) and lastly, in February 2021 (NL0221). The first two numbers of the sample code refer to the number of the month and the last two numbers, to the year (e.g. 0220 indicates February of 2020).

For microbial community analysis, 4 L of seawater was collected at Site B (Braided Nylon), at the bottom point (below the net) corresponding to control sample (no net) and on the surface (near the net) corresponding to fishing net sample. A total of 2 seawater samples per sampling campaign were collected. Sampling took place 3 h after low tide. Additional samples of seawater (1 L) were collected at each site (bottom/surface), for inorganic nutrient, total particulate matter, particulate organic matter and Chlorophyll a analysis with a sample bottle and transferred into a sterile plastic container. Afterwards, the nets were removed from the water, placed onto sterile trades and pieces of each net were collected for the biofilm microbial community analysis in all sampling campaigns, except at the beginning (February 2020). In March, additional pieces of each net were also collected for the cultivation of microorganisms in the laboratory. Physical-chemical parameters were taken in all sites and campaigns.

Once in the lab, the samples of nets collected for microbial community analysis were preserved at −80 ºC, and both the net biofilm samples collected for cultivation of microorganisms and the seawater samples were processed on the same day. For the microbial community analysis in seawater, 2 L of seawater was filtered through a 0.2 μm Sterivex™ filter attached to a manifold in a vacuum filtration system. Samples were concentrated in duplicate and Sterivex™ filters stored at −80 ºC until further microbial community DNA extraction.

### Environmental parameters and chemical characterization of seawater

An environmental and chemical characterization was made for seawater samples (surface and bottom) in every sampling campaign. Physical-chemical parameters, e.g. temperature, salinity, dissolved oxygen concentration, pH and turbidity, were measured on the site using a multiparametric probe (YSI EXO1 Sonde). Seawater samples (ca. 500 mL) were filtered through precombusted Whatman^®^ glass microfiber filters, Grade GF/F (Whatman, US) for total particulate matter (TPM) and particulate organic matter (POM) assessment. Afterwards, the filters were dried at 100 ºC (TPM) and then incinerated at 500 ºC (POM), following the protocol APHA^45^. For chlorophyll a analysis, ca. 500 mL of each seawater sample was filtered through cellulose acetate membrane filters (Whatman, US) (0.45 μm porosity). Filters were stored at −20 ºC until analysis. Chlorophyll a was determined spectrophotometrically after extraction with 90% acetone^46^ with cell/filter homogenization, using the^47^ trichromatic equation. The content of inorganic nutrients was analyzed in triplicate, from the previously filtrated water samples, through 0.45 μm filters. Dissolved concentrations of orthophosphate (PO_4_^3−^), nitrite (NO_2_^−^) and ammonium (NH_4_^+^ + NH_3_) ions were quantified according to Grasshoff et al.^48^ protocol, while nitrate (NO_3_^−^) ion was analyzed by an adapted spongy cadmium reduction technique^49^, subtracting nitrite from the total.

### Microbial community DNA extraction and next generation sequencing (NGS)

Total microbial community DNA of the seawater samples collected during the in situ experiment (Sect."In situ experimental set up and sampling*"*), was extracted using the PowerWater^®^ Sterivex™ DNA Isolation Kit, after defrosting the Sterivex™ filter units at room temperature. Regarding the DNA extraction for biofilm attached to the nets, cryopreserved net samples were unfrozen and total microbial community DNA was extracted using the DNeasy^®^ PowerSoil^®^ kit (Qiagen), following the vendors protocol, with minor modifications in the initial steps of the protocol: nets were cut in small transverse sections (ca. 2 cm) into 2 mL tubes, using UV sterilized scissors and tweezers; the solution present in the “PowerBead Tubes” was added to the 2 mL tubes followed by a 15 min centrifugation step (~ 10,000 rpm); the maximum of liquid and biomass, along with some net pieces, were transferred back into the “PowerBead Tubes” and the extraction continued as instructed in the vendor’s protocol.

The extracted DNA, of both seawater and net samples, were quantified with the kit Quant-it HsDNA in the Qubit fluorometer (Invitrogen), before sending to the biotechnological company Biocant – Biotechnology Park (Cantanhede, Portugal), where the total DNA was analyzed at the prokaryotic (Bacteria and Archaea) taxonomic level using metabarcoding next generation sequencing (NGS) technology. The hypervariable V6-V8 regions of the bacterial 16 S rRNA gene were amplified using the primers B969F (5′- ACGCGHNRAACCTTACC-3′) and BA1406R (5’-ACGGGCRGTGWGTRCAA-3′), developed by^50^. Pair-end sequencing was carried out in the Illumina MiSeq^®^ sequencer with the V3 chemistry (Illumina, Inc., San Diego, CA, USA) at Genoinseq laboratories.

Full protocol details are reported in Bragança et al.^51^. Raw reads extracted from Illumina MiSeq ^®^System were demultiplexed and pre-processed by the sequencing company; fastq files were quality-filtered with PRINSEQ (v. 0.20.4^52^;), to exclude sequence adapters, reads with low-quality Q25, and with a size of less than 100 base pairs.

### Data analysis for microbial communities

All the upstream and downstream data analysis for the microbial communities was performed in R program^53^ (R version 4.2.2).

#### Upstream analysis

The 16 S V6-V8 amplicon datasets are imported into R from the obtained demultiplexed fastq files and undergo the DADA2 pipeline where, in sum, sequences were quality filtered, trimmed, denoised and joined paired-end reads, providing at the end an Amplicon Sequence Variance (ASV) table as output^54^. Trimming of forward and reverse sequences was done at 270nt and 220nt, respectively, and 440760 reads from 6 samples were used for learning the error rates. After merging of the sequences, chimeras were removed and taxonomy was assigned to ASVs using the Silva rRNA (16 S SSU) database (version 138.1^55^,. Afterwards, eukaryotes, mitochondria, and chloroplast were eliminated from the 16 S dataset. The raw sequence data from this work is deposited to the European Nucleotide Archive (ENA) (Study accession number PRJEB62111).

#### Downstream analysis

With the obtained ASV table, alpha rarefaction curves were accessed and then, samples were normalized to the lowest number of reads present among samples (*n* = 4405) using the vegan package^56^. With the normalized community data, the alpha diversity indexes of Shannon, Berger-Parker (dominance) and the species richness (number of ASVs) were plotted, using the “ggplot2”^57^ and “grid” packages. For beta-diversity analysis, microbial communities were clustered in a dendrogram, based on Bray-Curtis distances, again with “vegan”, after applying a Hellinger transformation of data.

To assess and visualize differences between microbial communities, first comparing seawater samples with net biofilm samples, and then comparing net biofilms (from net A, B and C), non-metric multidimensional scaling (nMDS) was used. This multivariate statistical technique creates an ordination plot based on a dissimilarity or distance matrix and is commonly used in microbial ecology to interpret community structure. Then, the non-parametric statistical test ANOSIM (Analysis of Similarities) was employed, to compare the similarities within nets and between nets and seawater samples, based on distance measures, i.e., Bray-Curtis distances.

Taxonomic profiles of the seawater and biofilm bacterial communities were explored using the “phyloseq”^58^ package, with “tidyverse”^59^ to collapse taxa within a chosen a relative abundance threshold, “ggplot2”, “scales”^60^ and “reshape2”^61^ were used for graphics design, and “RColorBrewer” package^62^ for color association.

In this work, a search for potentially pathogenic microorganisms was performed in seawater and net biofilms, as well as potential pollutant-degrading microorganisms (hydrocarbons and plastic polymers). For that, the package “microViz”^63^ was employed after attributing a desired list of taxa. The list of potentially pathogenic genera used for the search was the following: *Clostridium*,* Arcobacter*,* Pseudarcobacter*,* Enterococcus*,* Chryseobacterium*,* Escherichia/Shigella*,* Lactococcus*,* Mycobacterium*,* Shewanella*,* Staphylococcus*,* Streptococcus*,* Aquabacterium*,* Blautia*,* Lactobacillus*,* Prosthecobacter*,* Reyranella*,* Iamia*,* Fluviivola*,* Paludibacter and Vibrio.* The genera *Pseudomonas* and *Acinetobacter* were not added to the pathogens list, as the authors consider these broad genera which hold environmental species with formerly reported potential for the degradation of pollutants such as hydrocarbons^64–67^. Regarding the hydrocarbon and plastic degrading list, the following genera names were chosen to look for: The OHCB marine genera *Algiphilus*, *Alcanivorax*,* Cyclocasticus*,* Oleiphilus*,* Oleispira*,* Planomicrobium*, *Polycyclovorans*,* Porticoccus* and *Thalassolituus*, and the genera *Acinetobacter*,* Alkanindiges*,* Altererythrobacter*,* Alteromonas*,* Bacillus*,* Colwellia*,* Dokdonia*,* Erythrobacter*,* Fabibacter*,* Flavobacterium*,* Glaciecola*,* Halomonas*,* Hyphomonas*,* Lutibacterium*,* Ideonella*,* Kocuria*,* Kordiimonas*,* Lewinella*,* Marinobacter*,* Marinobacterium*,* Methylophaga*,* Neptunomonas*,* Novosphingobium*,* Oleibacter*,* Pseudoalteromonas*,* Pseudomonas*,* Rhodococcus*,* Roseovarius*,* Shewanella*,* Sphingomonas*,* Sulfitobacter*,* Thalassospira*,* Winogradskyella*,* Yeosuana*^20,68–71^.

To distinguish bacterial families that were prevalent with net biofilms versus seawater matrices, we applied linear discriminant analysis (LDA) effect size (LEfSe)^72^, using the “microbiomeMarker” library^73^. Default values were used, except for LDA score which was 3.5.

### Isolation of biofilm-associated bacteria

The culture of bacterial strains associated with the fishing nets was conducted using one-month old net biofilm samples (NL0320). For that, pieces of each net were cut, placed into a 2 mL tube with saline solution (0.85%), submitted to a strong vortex step to promote the release of the attached biofilm and then, tenfold diluted. Pieces of each net, and the respective tenfold dilutions, were spread onto Marine Agar (MA) (CondaLab, Spain), Plate Count Agar (PCA) (Liofilchem, Italy) and Bushnell-Haas broth (BH) (Difco™) supplemented with 2% NaCl (v/v) and agar (15 g.L^−1^) and cultivated at 28 °C for 3–4 days. Once grown, morphologically different colonies were described and purified by the streaking method in the respective culture media. Biomass from each bacterial strain was collected and preserved in 20% glycerol at −80 ºC. Biomass was also collected for further DNA extraction and phylogenetic identification.

### DNA extraction and phylogenetic identification of bacterial strains

The DNA extraction of each collected bacterial strain was performed by using the E.Z.N.A.^®^ Bacterial DNA Kit (Omega Bio-Tek, GA, United States). After amplification of the V1- V9 regions of the bacterial 16S rRNA gene with the universal primers 27 F (5’ AGAGTTTGATCMTGGCTCAG 3’) and 1492R (5’ TACGGYTACCTTGTTACGACTT 3’), the amplified samples were separated by their molecular weight through a 1.5% agarose gel with SYBR Safe (Thermo Fisher Scientific, MA, United States). The resulting PCR products were sent for sequencing at the Genomics i3S Scientific Platform (Porto, Portugal). The 16 S rRNA sequences of both primers obtained for each strain, were aligned using the software Geneious (version 11.1.4), and the consensus sequences were blasted against those present in the nucleotide collection database of the National Centre for Biotechnology Information (NCBI), EZTaxon database (http://www.ezbiocloud.net) and Ribosomal Database Project (https://rdp.cme.msu.edu/). The 16 S rRNA gene sequences of the identified strains attached to each net’s biofilms, were placed in GenBank (NCBI, Maryland, USA) under the accession numbers indicated in Table S1 of the supplementary material.

## Results

### Environmental and chemical characterization of seawater

Visually, biofouling on the nets occurred after just one month in seawater, where not only the growth of microbial biofilm was observed, but also the beginning of the attachment of eukaryotic organisms such as algae and mussels. The overall succession of biofouling onto nets, throughout the experiment, is shown in Figure S1 (supplementary material). Thin Nylon net was the one in which biofouling occurred faster, showing after just one month the growth of macroalgae and bivalves. In the other nets, macroalgae and bivalve growth was only observed after 8 months of experiment (October 2020 (1020)). This biofouling increased with time, increasing biomass and weight in the nets.

The physical-chemical parameters throughout the experiment can be found at Table S2 (Supplementary materials), while the concentrations of inorganic nutrients, chlorophyll a and particulate matter in surface and bottom seawater samples are shown in Table S3 (Supplementary materials).

Chlorophyll a concentrations were on average 2.16 µg L^−1^, but higher in July 2020 (0720, ca.3.70 µg L^−1^) and October (1020, 5.04 µg L^−1^) samplings. Total particulate matter values varied little amongst sites and seasons, ranging from 0.03 mg L^−1^ to 0.15 mg L^−1^. Regarding nutrient analysis, overall concentrations were higher in surface seawater (surrounding the nets) than in bottom seawater, where generally NO_3_^−^ > NH_4_^+^ > PO_4_^3−^ > NO_2_^−^. Nitrate ions (NO_3_^−^) concentration were higher in the winter samplings of 0220 (ranging from 35 to 120 µM L^−1^) and 0221 (ranging from 40 to 134 µM L^−1^), compared to an average of 24.7 µM L^−1^ for the remaining sampling times. Nitrite ions (NO_2_^−^) concentrations, on the other hand were higher at the beginning of the experiment (0220, ~ 2 µM L^−1^ on average) compared to the rest of the experiment (~ 1 µM L^−1^ on average). Ammonia was higher at 0220 (17.6 µM L^−1^ on average) and at 0520 (~ 16 µM L^−1^ on average) sampling times, compared to the rest (~ 7.35 µM L^−1^ on average). Phosphate ion concentrations varied from 0.5 to 3.2 µM L^−1^, presenting higher values at 0520.

### Alpha-diversity of microbial communities in seawater and biofilms

Next-generation sequencing of the 27 samples collected in the in situ experiment, generated a total of 2,029,118 sequences of 16 S rRNA gene. The number of raw sequences and sequences filtered throughout the DADA2 pipeline for the 16 S amplicon datasets is summarized in Table S4 (supplementary material).

**Fig. 2:**
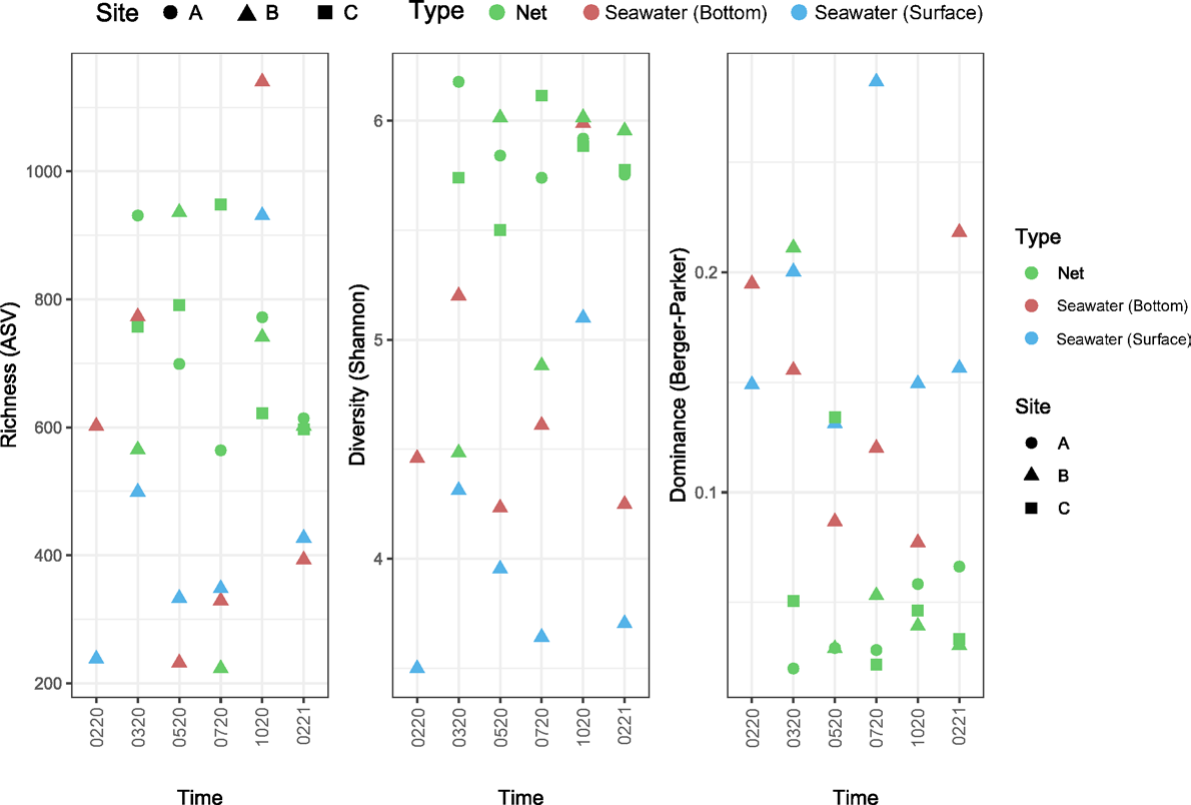
Alpha diversity analysis of the microbial communities from the seawater (surface and bottom) and net biofilm samples Braided Polyethylene (PE) (Site A), Braided Nylon (Site B) and Thin Nylon (Site C), collected from the in situ experiment at marina of Leixões, Matosinhos from left to right: Number of observed ASVs (Richness) Diversity (Shannon index) and Dominance (Berger Parker index). Seawater was collected at all sampling times: 0220 (fev_2020), 0320 (mar_2020), 0520 (may_2020), 0720 (jul_2020), 1020 (oct_2020) and 0221 (fev_2021), while net samples were collected at all sampling times, except at the beginning (0220).

Afterwards, the diversity of the microbial communities in seawater (surface and bottom) and net biofilms samples was assessed (Fig. 2). The plot of the alpha rarefaction curves (Figure S2, supplementary material) suggests a good sequencing effort for most samples and a fair representation of their microbial communities, with the reaching of plateau level for the observed ASVs. The samples NLBR0720 and NLBF0720 reached a plateau, however they presented lower sequencing depth.

Overall, microbial communities in net biofilms were more diverse than the communities from seawater. Species richness of all communities ranged from 200 to 1200 observed ASVs for all samples and was generally higher in biofilm communities.

Seawater communities at October 2020 (1020) had higher number of observed ASVs compared to net samples at that time and other seawater samples, however, when it came to diversity, biofilm communities at 1020 were similar to the ones at the bottom (seawater). Being mostly less diverse than nets, seawater samples presented more dominant taxa, indicating a more selected community here, except for net sample in March (NLBR0320) and July (NLCR0720).

### Structure of the microbial communities

**Fig. 3:**
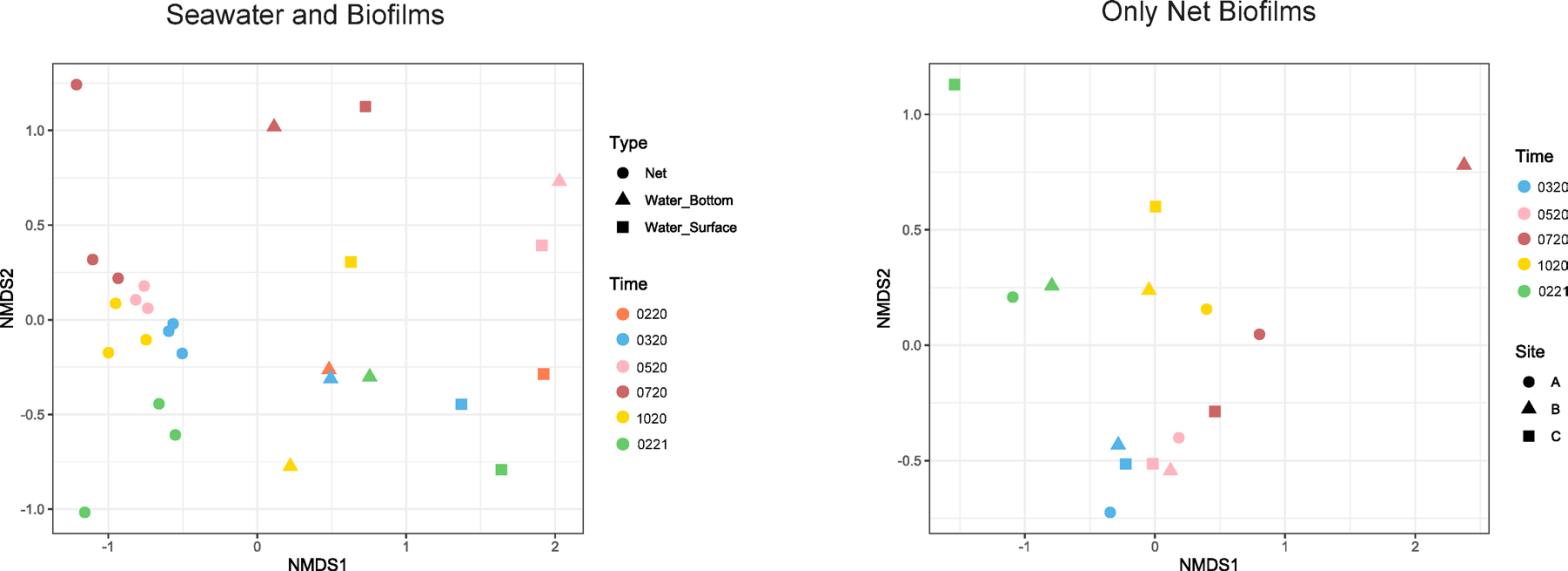
Multidimensional Scaling (NMDS) of the seawater (surface and bottom) and net biofilm samples Braided Polyethylene (PE) (Site A), Braided Nylon (Site B) and Thin Nylon (Site C), collected from the in situ experiment at marina of Leixões, Matosinhos, at all the sampling times 0220 (fev_2020), 0320 (mar_2020), 0520 (may_2020), 0720 (jul_2020), 1020 (oct_2020) and 0221 (fev_2021).

The beta-diversity analysis of the seawater and net biofilm microbial communities is displayed in Fig. 3. Based on NMDS and analysis of similarities (ANOSIM) analysis, we can confirm that microbial communities from seawater were significantly different from those of net biofilms (*R* = 0.9839, *p* = 0.001). Regarding only net biofilms, there was an apparent temporal succession, shown by a proximity of the communities from continuous sampling times and while polymer type did not influence the microbial community composition, sampling time did (ANOSIM, *R* = 0.7437, *p* = 0.001).

Hierarchical clustering, by a dendrogram, separated the communities of seawater and biofilm samples into two main groups (Figure S3). Within the cluster of the nets, samples collected in 2020 are clustered together, and distinct from the ones collected after 1 year of experiment, in February 2021 (0221). The biofilm communities of the 3 nets cluster together within the same sampling time, being samples from march and may closer to each other, than to July and October samples. The sample of Net B from the July sampling campaign (NLBR 0720) appears on a branch apart from the other net samples of this month. This might be the consequence of the low sequencing effort obtained for the latter sample (Figure S3). Regarding seawater communities, within the sampling times of 0520, 0720 and 1020 surface (NLBS) and bottom (NLBF) communities are similar and cluster together. Separated in two more distant clusters, are the microbial communities from winter season, 0220, 0320 and 0221.

These results might suggest a succession of the net biofilm communities throughout the experimental time, independent of their polymer type, which could have been influenced by seasonal changes, biofilm maturation or other environmental factors, with season-driven variations if considering the free-living microbial communities in seawater.

### Microbial community composition

**Fig. 4:**
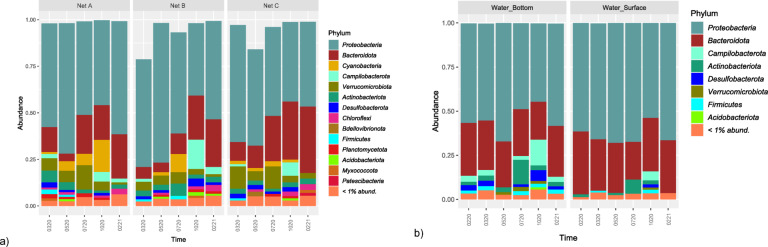
Taxonomic composition at the Phylum level of microbial communities present in both (a) nets (Net A - Braided Polyethylene (PE) (Trawling net), Net B - Braided Nylon (Seine net) and Net C - Thin Nylon (Seine net) and (b) seawater samples, collected from the in situ experiment of marina of Leixões. Seawater samples were collected at all sampling times (0220 (fev_2020), 0320 (mar_2020), 0520 (may_2020), 0720 (jul_2020), 1020 (oct_2020) and 0221 (fev_2021)while net samples were collected at all times except for 0220 (fev_2020). Phylum composition of groups with more than 1% relative abundance in the communities. Graphs on the left belong to net biofilm samples, while graphs on the right belong to seawater samples.

**Fig. 5:**
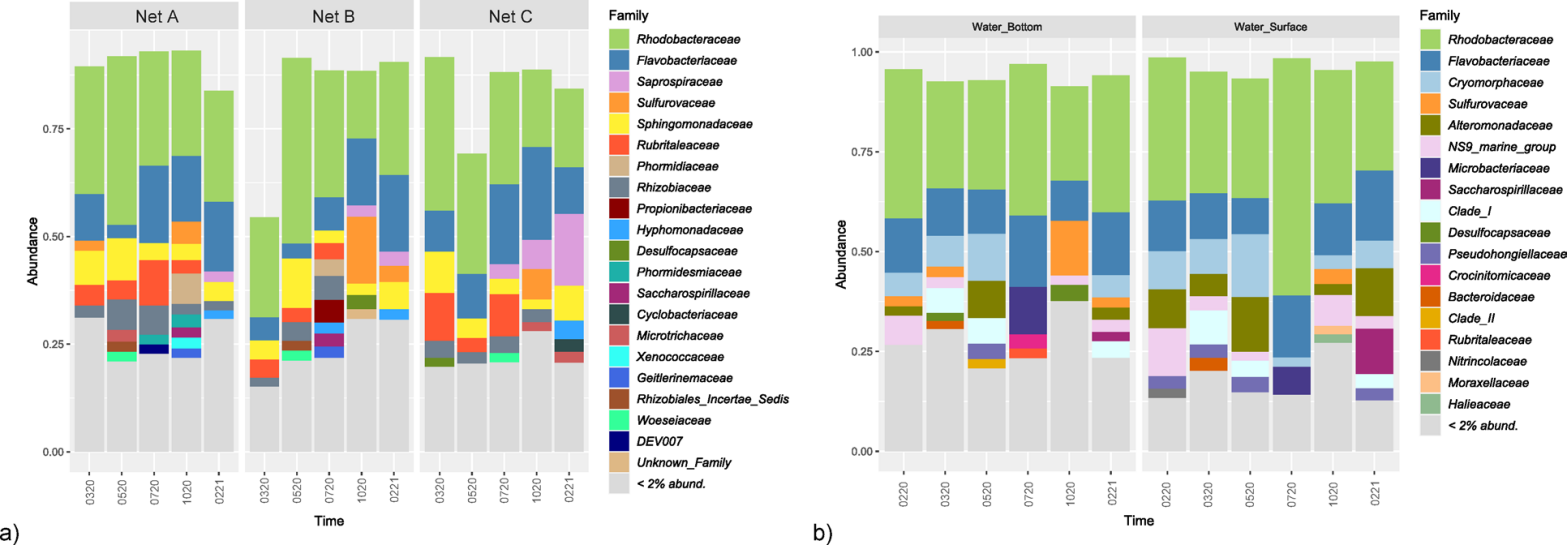
Taxonomic composition at the Family level of microbial communities present in both (a) nets (Net A - Braided Polyethylene (PE) (Trawling net), Net B - Braided Nylon (Seine net) and Net C - Thin Nylon (Seine net) and (b) seawater samples, collected from the in situ experiment of marina of Leixões. Seawater samples were collected at all sampling times (0220 (fev_2020), 0320 (mar_2020), 0520 (may_2020), 0720 (jul_2020), 1020 (oct_2020) and 0221 (fev_2021) while net samples were collected at all times except for fev_2020.; Family composition of groups with more than 2% relative abundance in microbial communities. Graphs on the left belong to net biofilm samples, while graphs on the right belong to seawater samples.

The taxonomic structure is represented in Figs. 4 and 5, at the Phylum (> 1% abundance) and Family (> 2% abundance) level, respectively.

#### Free-living communities in seawater

Bacteria from the phyla Proteobacteria and Bacteroidota (or Bacteroidetes) were the most dominant phyla of the seawater communities, where Proteobacteria represented more than half of the community in all samples except for NLBF1020 and NLBF0720 (Fig. 4). The dominance of these two phyla was more accentuated in the winter months (0220, 0320, 0221) and spring (0520) samples of surface seawater. During winter, the phyla Campilobacterota, Actinobacteriota (or Actinobacteria), Desulfobacterota and Firmicutes were observed in the communities of bottom seawater samples but not on the surface communities from that time. On the autumn seawater samples (1020) Campilobacterota (~ 5% NLBS1020, ~ 15% NLBF1020) and Desulfobacterota (~ 2% NLBS1020, ~ 6% NLBF1020) phyla were the most represented. Actinobacteriota members were more abundant in summer seawater samples (NLBS0720, ~ 8% and NLBF0720, ~ 14%). Within seawater communities, the families *Rhodobacteraceae*, *Flavobacteriaceae*, *Cryomorphaceae Sulfurovaceae*, *Alteromonadaceae* and *Microbacteriaceae* were the most representative (Fig. 5).

#### Plastic Nets associated biofilms

Similar to seawater communities, Proteobacteria (*Rhodobacteraceae* family) and Bacteroidota (*Flavobacteriaceae* family) dominated the nets biofilms, yet an increase of Bacteroidota abundance was observed in more mature biofilms, after 5 months of incubation from ~ 7% to ~ 19% (0720), ~ 24% (1020) and ~ 28% (0221) average relative abundances, consistent with the abundances increase of *Flavobacteriaceae and Saprospiraceae* families (Fig. 5). Bacteria from Verrucomicrobiota phylum were more abundant in net biofilms, scattered through all polymers and time (from ~ 1% to ~ 13% abundances). In this work we observed correlation only with the biofilm communities (above 1% abundance), the phyla Cyanobacteria, Chloroflexi, Planctomycetota, Bdellovibrionota, Myxococcota and Patescibacteria (Fig. 4).

**Fig. 6:**
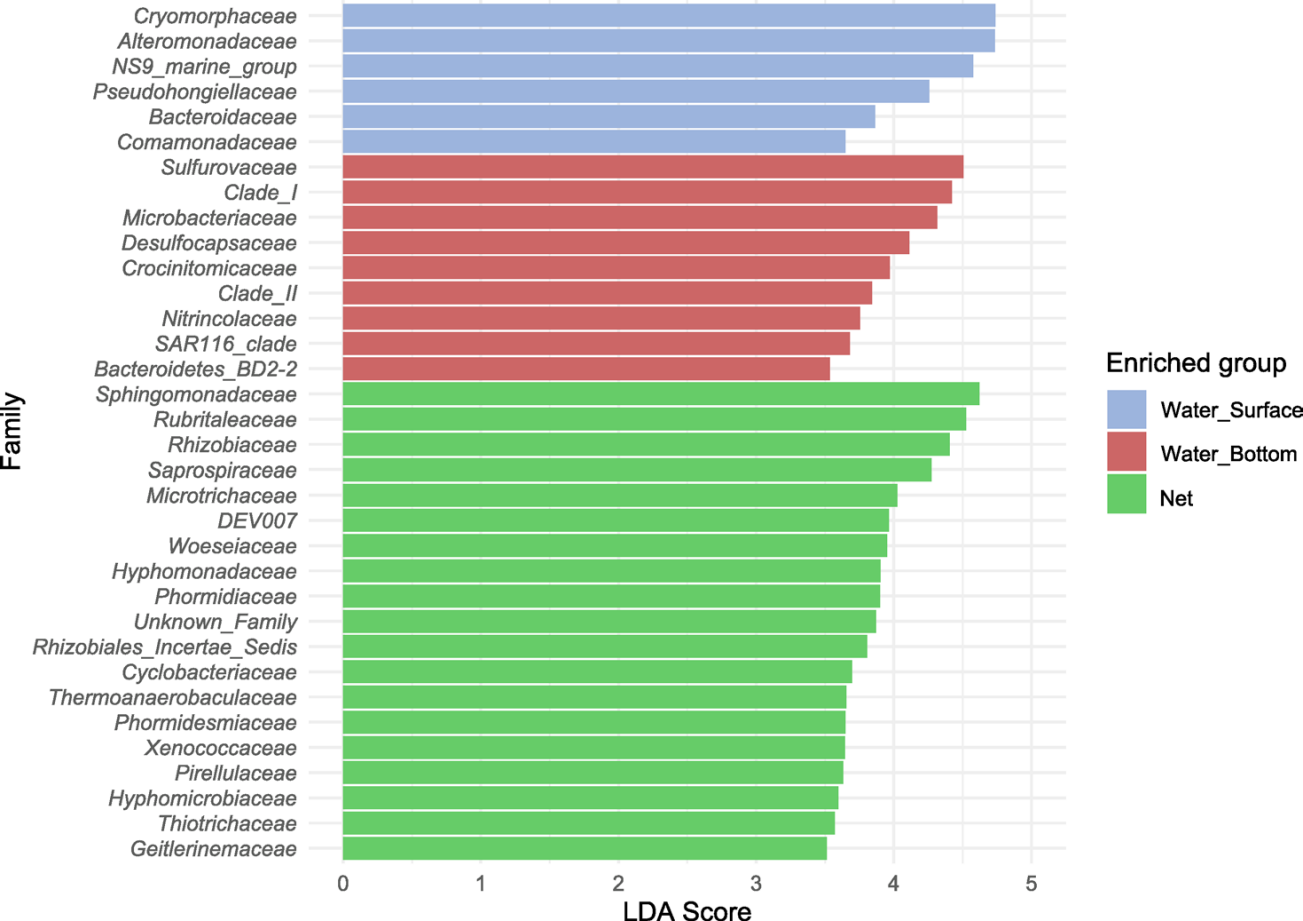
LEfSe analysis identifying prevalent taxa (LDA score > 3.5) on seawater (Water Surface, Water Bottom) and net biofilm bacterial communities.

LEfSe analysis (Fig. 6) illustrated the taxon-specific differences between seawater and net matrices. The families *Sphingomonadaceae*, *Rubritaleaceae*, *Rhizobiaceae* and *Saprospiraceae* were found to be discriminative of net biofilms, while *Cryomorphaceae*, *Alteromonadaceae*, *Sulfurovaceae* and Clade I families were representative of the seawater communities.

#### Detection of potentially pathogenic microorganisms in net biofilms

**Fig. 7:**
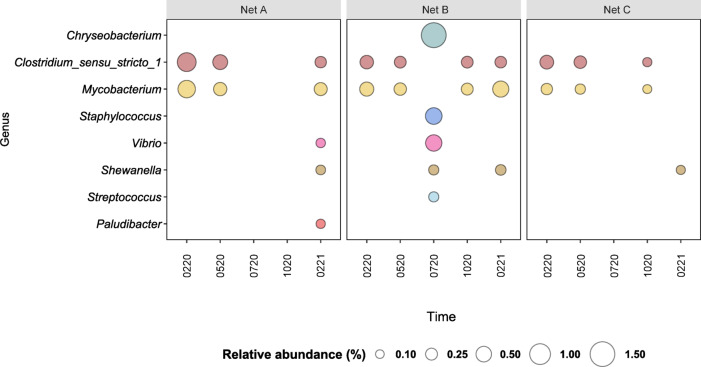
Bubble plot showing the relative abundance (%) of potentially pathogenic genera present in the communities (above 0.1% in relative abundance) of net biofilm samples collected from the in situ experiment of marina of Leixões, at the sampling times 0320 (mar_2020), 0520 (may_2020), 0720 (jul_2020), 1020 (oct_2020) and 0221 (fev_2021).

A search for a list of potentially pathogenic taxa (above 0.01% in abundance) was performed for the net biofilms, throughout time (Fig. 7). Most of the potentially pathogenic microorganisms were below 1% abundance in all nets and sampling times, except for *Chryseobacterium* genus in the Net B (Braided Nylon) after 5 months of experiment (NLBR0720),

Initially, the fishing net biofilms (0320 and 0520) comprised only the potential pathogenic genera of *Clostridium_sensu_stricto_1* and *Mycobacterium*. After 5 months of experiment (0720), potential pathogenics were only found in Net B, such as *Chryseobacterium* (~ 1.5%), *Staphylococcus* (~ 0.6%), *Streptococcus* (~ 0.2%), *Vibrio* (~ 0.6%) and *Shewanella* (~ 0.2%). Afterwards, the genera of *Clostridium_sensu_stricto_1* and *Mycobacterium* reappear in the nets B and C in October 2020 (1020). In the one-year-old biofilm of nets A and B, we can see again both *Clostridium* and *Mycobacterium* above 0.1% abundance, but also the genera of *Shewanella* (for both nets), *Vibrio and Paludibacter* (for Net A). In Net C, only *Shewanella* was detected in the 1-year biofilm.

When counting the abundance of all pathogenic genera in the net biofilms, their combined abundance was mostly below 1.5% except in Net B at 0720, that reached ~ 3.2% of the community.

Regarding the presence of pathogens in the seawater, represented in Figure S4 (supplementary materials), a higher diversity and abundance of these genera was found in the bottom seawater samples, comparatively to the surface seawater samples on the respective sampling time. Each genera alone represented less than 1% of the community, where the genera of *Mycobacterium*, *Vibrio*, *Clostridium*, were the most represented. In the bottom seawater samples *Escherichia/Shigella* and *Staphylococcus* appeared on 0520 while in the Net B biofilm these genera only appear on the next sampling time: 0720. The genera of *Blautia* and *Aquabacterium* only appeared in surface seawater samples at 1020 (below 0.2%) and the *Arcobacter* only in seawater samples after 1 month (0320) and 1 year (0221) in bottom samples.

The detection of certain genera, especially *Mycobacterium* and *Clostridium*, in the bottom seawater samples and in nets biofilm, might suggest a slight accumulation of these pathogens in the nets. However, they are detected simultaneously in biofilms and seawater samples at 1020 and 0221. Curiously, for both biofilms and seawater, when *Staphylococcus* prospered (along with *Escherichia/Shigella* for the seawater samples), the genera *Mycobacterium* and *Clostridium* decrease their abundance (below 0.1%). Overall, the abundance of pathogens remained stable in the net biofilms, and seasonal variations did not seem to impact the seawater matrices.

#### Detection of hydrocarbon and potentially plastic degrading taxa in net biofilms

**Fig. 8:**
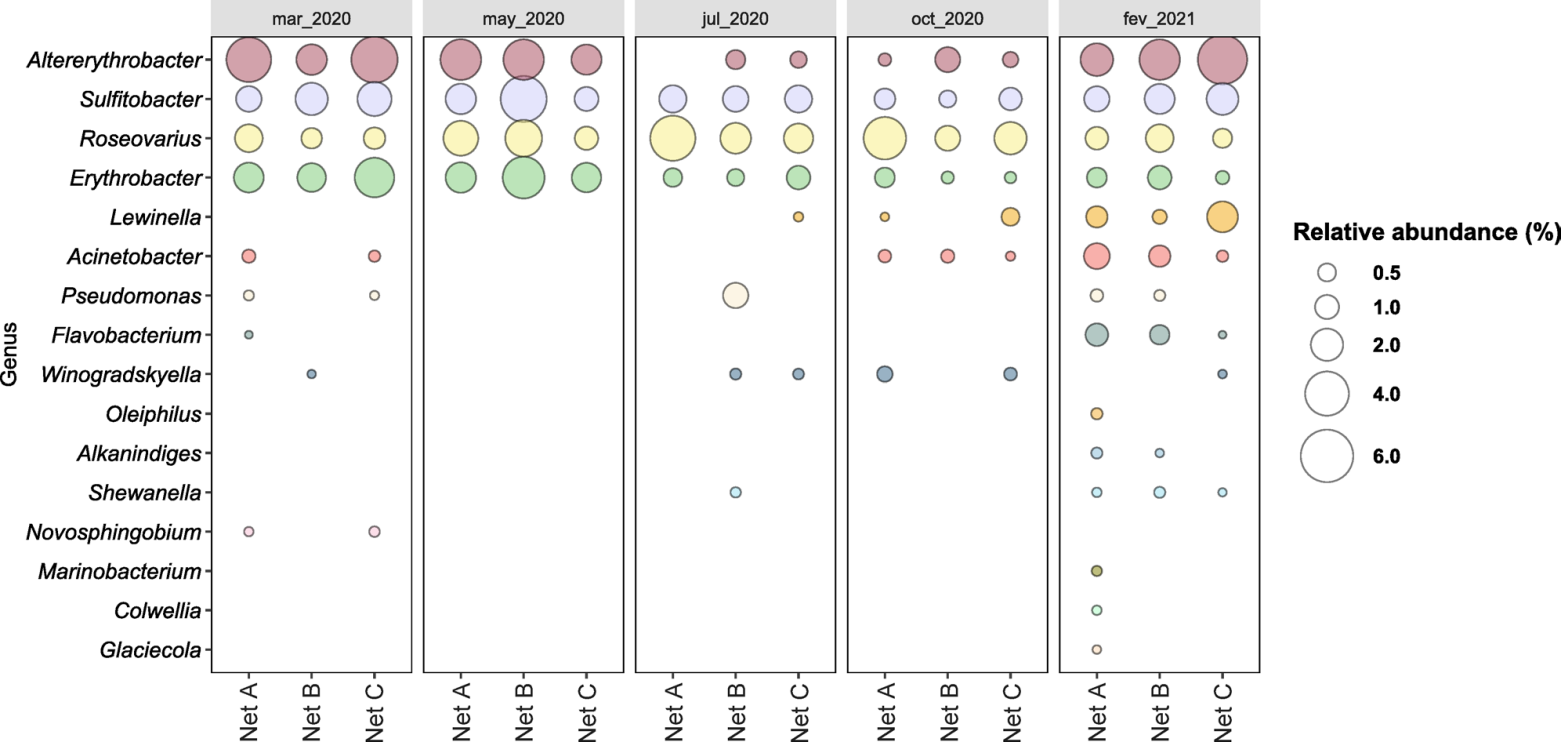
Bubble plot showing the relative abundance (%) of Bacterial genera with members previously reported for their hydrocarbon and/or plastic degrading capacity, present in the communities (above 0.1% in relative abundance) of net biofilm samples (Net A - Braided PE, Net B - Braided Nylon and Net C - Thin Nylon) collected from the in situ experiment of marina of Leixões, at the sampling times 0320 (mar_2020), 0520 (may_2020), 0720 (jul_2020), 1020 (oct_2020) and 0221 (fev_2021).

By searching for potentially hydrocarbon or plastic degrading genera (Fig. 8) we can observe the presence of 16 genera above 0.1% in abundance. *Altererythrobacter*, *Sulfitobacter*, *Roseovarius*, *Erythrobacter* were always highly represented in the 3 nets biofilm communities over time, being the only potential degraders at the 3-month-old biofilms (0520).

On the one-year-old biofilms (0221) a higher diversity of hydrocarbon-degrading genera, present above 0.1%, was evident. The overall joined abundance of hydrocarbon/plastic degrading genera from the beginning (one month, ~ 9% for NLAR0320, ~ 6% for NLBR0320, ~ 11.3% for NLCR0320) to the end of the experiment, after 1 year incubation (~ 8.8% for NLAR0221, ~ 9.6% for NLBR0221, ~ 10.4% for NLCR0320) was similar in nets biofilms. Overall, Braided PE (Net A) sheltered a higher diversity of degraders, followed by Braided Nylon (Net B).

### Culturable bacterial strains identification and their representation in biofilm communities

A total of 122 bacterial strains were collected from the net biofilms and identified, comprising a variety of 44 different bacterial genera. The closest phylogenetic identification, as well as the NCBI accession numbers of each strain, are indicated in Table S1 (Supplementary materials). Bacteria were mostly from the class Gammaproteobacteria (Proteobacteria phylum; 42 strains), Actinomycetia (Actinomycetota phylum; 35 strains) and Bacilli (Bacteroidota phylum; 21 strains). Most of the recovered genera correspond to the isolation of one bacterial strain. Figure S5 (in supplementary materials) shows the number of bacterial genera isolated more than once in each net (Figure S5a) as well as the distribution of bacterial genera among nets, via a Venn Diagram (Figure S5b).

Overall, strains of *Acinetobacter*,* Bacillus*,* Rhodococcus*,* Shewanella*,* Streptomyces* and *Vibrio* were common to all nets, with Braided Nylon (Net B) showing the highest diversity of bacteria (26 genera in total, where 17 were specifics to this net), followed by Thin Nylon (Net C) and Braided PE (Net A), both with 23 genera. Most of the recovered genera belonged to *Acinetobacter* (7 strains), *Bacillus* (9 strains), *Microbacterium* (7 strains), *Paraglaciecola* (8 strains), *Rhodococcus* (9 strains), *Shewanella* (7 strains) and *Streptomyces* (10 strains) genera.

**Fig. 9:**
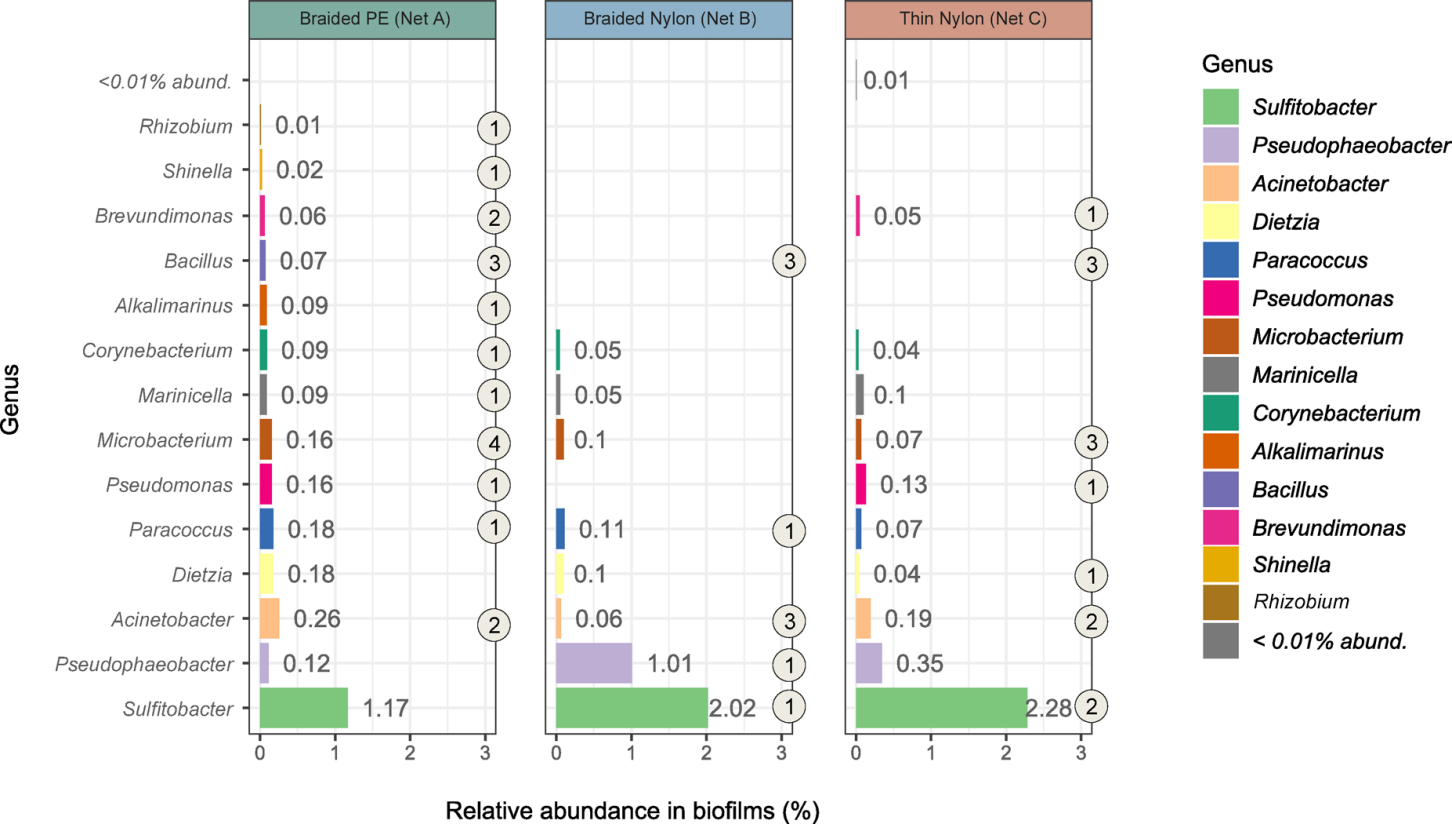
Barplots representing the relative abundance (%) of bacterial genera found in the one-month-old biofilm communities (0320, above 0.01% abundance) and cultured in the lab, from net samples collected in the in situ experiment of marina of Leixões (Net A - Braided PE, Net B - Braided Nylon and Net C - Thin Nylon). Circles indicate the number of bacterial strains isolated from the respective genus and net.

When comparing the isolated bacterial strains with the microbial community present in the one-month-old net biofilms, few were the genera representative of the communities (above 1% relative abundance), namely *Sulfitobacter* and *Pseudophaeobacter.* Instead, 13 genera (*Acinetobacter*, *Dietzia*, *Paracoccus*, *Pseudomonas*, *Microbacterium*, *Marinicella*, *Corynebacterium*, *Alkalimarinus*, *Bacillus*, *Brevundimonas*, *Shinella* and *Rhizobium*) were found between 0.01 − 0.3% (Fig. 9). These results indicate a poor success rate in the recovery of the dominant bacterial genera in the communities of 0320 (between 2 − 10% relative abundance), such as *Rubritalea*, *Silicimonas*, *Yoonia-Loktanella* and *Altererythrobacter* (Figure S6, supplementary materials).

## Discussion

Intending to study the bacterial communities associated with plastic fishing nets, the latent pathogenic risks and hydrocarbon and plastic- degrading potential, the microbial community succession in 3 plastic fishing nets (made of High-Density Polyethylene and Nylon 6.6 polymers) submerged in seawater was monitored, in an in situ experiment at a marina. In addition, the isolation of bacterial strains from net biofilms was also done in the laboratory. The two types of polymers chosen for this study (Polyethylene and Nylon 6,6), are amongst the most used in fisheries^74^, but also part of the most abundant plastic polymer types found in aquatic environments, reported in a recent meta-analysis conducted by Erni-Cassola et al.^75^.

After being submerged in seawater, the fishing nets quickly developed a microbial biofilm and served as substrate for biofouling of other organisms, such as algae and bivalves, as is commonly observed for fishing gear and boats^7,76^. The Thin Nylon net, used in seine fisheries, with a monofilament of 0.3 mm in diameter, was the one that demonstrated a faster and the largest biofouling of macroorganisms. When lost in the ocean, monofilament fishing lines can inflict hurt or even cause the death of marine wildlife such as dolphins^77^, fruit of the entanglement in the net.

### Microbial communities’ succession

There are numerous factors that can drive community changes in biofilms attached to plastic surfaces, like environmental conditions, location, type of and weathering state of plastic polymers^20,23,24^. It was no surprise when, in this work, communities from seawater matrices differed from those attached to net biofilms, as it has been continuously reported by previous studies^19,22,78^. In our in situ experiment, free-living microbial communities in seawater varied according to season, as also observed for seawater physico-chemical parameters, contrary to the net biofilms, where our results suggest a temporal succession regardless of their polymer type, which we hypothesize could result from biofilm maturation, seasonal changes, or other environmental factors such as nutrient concentrations and biotic interactions with other organisms from the site. Different results were seen in former studies, where communities differed among polymers^21,23^.

An in situ experiment conducted for 30 days at two marinas (Sea of Oman), where pieces of polyethylene terephthalate (PET) and polyethylene (PE), but also non-plastic substrates, like steel and wood, were submersed in seawater^23^ observed that communities varied between substrates (plastics were different from wood or steel) and also location. Still, the authors point out that substrates differed on OTUs that represented less than 3% of relative abundance.

Pinto et al.^21^ also evidenced plastic-type specific taxa after an in situ incubation, this time in the Adriatic Sea, though differences among polymers (HDPE, LDPE and PP) were more noticeable at initial stages of colonization (one week) rather than at later stages (one and two months). At later stages, general biofilm processes took place and all polymers revealed high abundances of families such as *Flavobacteriaceae*,* Rhodobacteraceae* and *Planctomycetaceae*. In our study *Flavobacteriaceae* and *Rhodobacteraceae* families were very abundant not only on net biofilms but also on seawater samples, after one and two months of experiment.

In the present study, we did not introduce a control surface like glass, nor did we analyze initial steps of microbial community colonization, before one month of the experiment. Future characterization of initial stages of microbial colonization on fishing nets, might be thought of.

Few are the experiments looking into the microbial communities establishing on fishing gear^43,44^. Aiming to compare the bacterial microbiomes, and identify potential pathogens associated with nylon (an old 5-year net, and a new one) and copper net pens, Canada et al.^43^ conducted an in situ experiment inside a fish farm in Madeira, for 125 days. The authors observed similar taxonomic profiles at phylum level, with the dominance of Proteobacteria (> 30%) and Bacteroidota (or Bacteroidetes, > 15%) like in our study (between 39 − 75% and 4 − 36%, respectively), but also Planctomycetota (or Planctomycetes, ~ 20%), which in our study were present in lower abundance in nylon nets (~ 1.2%). On the genus level, the genera *Altererythrobacter* (new net), *Ruegeria* (both) and *Winogradskyella* (old net) also exhibit > 1% abundance. Although with marine coastal water, the mention research was performed within populated fish tanks, since the purpose of the study was aligned with aquaculture.

De Tender et al.^44^ on the other hand, compared microbial communities growing on plastic PE samples, such as transparent plastic sheet and orange-colored dolly ropes (1 mm monofilament), inside a harbor (and an offshore point) located in the Belgian part of the North Sea. The authors observed that sample type (sheet, dolly rope, seawater, or sediment) location and sampling time significantly shifted the microbial communities. While sampling during the first month and then, monthly up to 10 months of experiment, showed a gradual change in bacterial community composition on plastic samples collected from the harbor, more noticeable differences between plastics were observed in the early steps of colonization, like in Pinto et al.^21^.

In the current study, the families *Sphingomonadaceae*, *Rubritaleaceae*, *Rhizobiaceae*, *Saprospiraceae* and *Hyphomonadaceae*, were observed to be discriminative of plastic net biofilms, which was also observed in enriched on plastic surfaces of other studies^27,79^. Correlated more with seawater communities, we detected the families *Cryomorphaceae*, *Alteromonadaceae*, *Sulfurovaceae* and *Clade I* families. Curiously both *Cryomorphaceae*, *Alteromonadaceae* are commonly discriminating on plastic communities, like PET, in other studies^21,80^.

### Choice of 16 S rRNA gene regions for sequencing

In our study, the V6-V8 regions of the 16 S were amplified and sequenced to evaluate the microbial communities in both biofilms as in seawater samples. The choice of different amplicons of the 16 S can influence the final taxonomic results on the microbial communities. Studies on microbial communities attached to plastic particles usually use V3-V4^44,81^ V3-V5^78^, V4^27^ or V4-V6 regions^21^ of the 16 S rRNA gene and marine microbiomes studies employ the sequencing of the V4-V5 regions, instead^82^. The usage of the V6-V8 regions, although specific for bacteria, might lead to the underestimation of Archaea taxa, compared to V4-V5, nonetheless it can also lead to the increase of rare bacterial taxa coverage^83^, ecologically important in microbial community dynamics^84^. This greater coverage of the rare biosphere is advantageous, as plastic-specific microorganisms have been found to be related to members of the rare biosphere within biofilms attached to plastic^26^. Nevertheless, in this study, the same regions were sequenced for either seawater or net biofilm samples.

### Pathogenic communities in seawater and fishing Nets biofilms

The idea that microplastics and plastics in the marine environment could act as a transportation vector for pathogens still generates questions and interest in research, ever since the description of the “plastisphere” by Zettler et al.^19^. The work of Marques et al.^85^ evidenced the presence of potential pathogenic genera like *Staphylococcus*,* Mycobacterium* and also bacterial genera correlated with WWTP/sewage (i.e., *Blautia*, *Lactobacillus*, *Lactococcus*) to be specific on the collected plastic particles from the Mondego River estuary and 3 adjacent beaches, in Portugal. Kirstein et al.^13^ demonstrated the presence of cultivable *Vibrio* spp. (at the species level, via MALDI-TOF MS technique), in microplastics collected from the North and Baltic Sea. However, the authors point out the co-occurrence of these taxa in the surrounding seawater, as well. In our study, *Vibrio* was not a notorious pathogen, since it represented less than 1% in seawater samples and was only detected in Braided PE (Net A) at 0221 and Braided Nylon (Net B) at 0720, again below 1% in relative abundance.

The comparative reanalysis of Oberbeckmann and Labrenz^29^ pointed out that microplastics alone did not correspond to higher risks of pathogens transport nor accumulation, since the median relative abundances of potentially pathogenic taxa (members of *Arcobacter*, *Pseudomonas*, *Shewanella*, and *Vibrio*) in microplastics were below the ones associated with natural control surfaces like wood or glass and particle-attached water fraction. Nevertheless, they stress the higher durability of plastics, and the lower degradability when compared with natural surfaces, which could lengthen the distances and time for which the pathogens are carried across the ocean.

#### But can the same risks be associated with ghost fishing nets?

In our study, we detected potential pathogenic genera like *Clostridium* and *Mycobacterium* in most biofilm samples throughout time. The detection of the previous genera in the bottom seawater samples and nets, but not on the surface, might suggest a slight influence of the seawater in the distribution of these pathogens in fishing nets. Still, they were present in both biofilms and seawater samples later on the experiment (after 9 months and one year). Notably, individually, the abundances of each potential pathogenic genus were always below 1% for seawater samples and 1.5% for net biofilms, respectively, with no accumulation of these taxa detected over time. As previously mentioned, plastic litter items can be a potential vector of pathogens across the ocean, and given the slight tendency observed in this study for fishing nets to adsorb potential pathogenic taxa, we cannot rule out the possibility of plastic fishing nets to act as carriers as well, when drifting away to more pristine areas.

### Hydrocarbon/plastic-degrading potential in biofilm-associated communities

When analyzing the communities attached to different microplastics polymers, Debroas et al.^35^ observed the enrichment in metabolisms involved in xenobiotic degradation when compared to the surrounding seawater, namely for the degradation pathways of Chlorocyclohexane and chlorobenzene, Polycyclic aromatic hydrocarbons and Nitrotoluene. Bryant et al.^28^ observed the enrichment of xenobiotic degradation pathways as well, in plastics recovered from the north Atlantic gyre. This gives a hint to the potential xenobiotic degradation occurring in plastic biofilms.

But could this degradation be addressed for the pollutants adsorb to plastic and microplastics or could there also be pathways for polymers degradation there, unknown so far?

In the net biofilm communities from our study, there was a continuous presence of the hydrocarbon-degraders like *Altererythrobacter* (up to ~ 5%), *Sulfitobacter* (up to ~ 4%), *Roseovarius* (up to ~ 4%) and *Erythrobacter* (up to ~ 3.5%) genera, in all nets across time. The works of Curren and Leong^86^ and Dussud et al.^78^ highlight the dominance of *Erythrobacter* members (around ~ 21% and ~ 43%, respectively) in bacterial communities associated to micro- and macro- plastic marine debris, collected either along the coastline of Singapore or in the Mediterranean Sea. Studies like Bryant et al.^28^ and Pollet et al.^87^ reported also a dominance of hydrocarbonoclastic bacteria in the communities associated with fossil-based plastics.

Unlike the previously mentioned studies, the potential hydrocarbon or plastic genera did not dominate the biofilm communities in our work (ranging from 3% to a maximum of 14% of the total biofilm community per sample). Although after 1 year, a higher diversity of hydrocarbon-degrading genera, present above 0.1%, was evident in the net biofilms, although their overall relative abundances did not vary from the early colonization stages of the fishing nets (~ 9% on average).

In addition, the obligate hydrocarbonoclastic bacteria (OHCB) *Oleiphilus* and *Porticoccus* were found in the 1-year biofilm of Braided PE net, although in low abundances (between 0.1% and 0.2% in abundance). Members of OHCB have been reported enriched in plastic biofilms from ocean marine debris or laboratory experiments^68^ and even implied in the degradation of some plastic polymers, as is the case of *Alcanivorax borkumensis* implicated in the degradation of LDPE^81^.

Recently, members of *Oleiphilus* were reported to be enriched on weathered (5.8%) and non-weathered polyethylene (3.7%), after in situ incubations in coastal seawater, as well as the enrichment of other hydrocarbonoclastic bacterial genera like *Roseobacter* and *Aestuariibacter* in the work of Erni-Cassola et al.^24^. Yet, it occurred during early stages of colonization (after just 2-days), whereas in our work it happened in the mature biofilm of Braided PE net, after one year, in much lower abundance.

This greater representation of hydrocarbonoclastic taxa in early stages of plastic colonization (from 8 to 35% of the total OTUs) compared with mature biofilms (from 6 to 12% of the total OTUs) was also stated in the work of Jacquin et al.^88^, where the biofilm formation was observed for conventional (PP) and “biodegradable” materials (i.e., PLA and PBAT), immersed in seawater for 40 days, following laboratory experiments until 94 days of incubation with each material in minimal media.

### Culturable bacteria in net biofilms and their representation communities

Overall, most of the bacterial strains cultivated in the lab, from net pieces collected one month after placing the fishing nets in the seawater, were not representative of the respective biofilm communities (> 1% in abundance), except for *Sulfitobacter* and *Pseudophaeobacter*. In fact, those two genera were recovered from the net biofilms in which they were most abundant on (*Sulfitobacter* from Thin nylon, and *Pseudophaeobacter* from Braided Nylon net). Instead of justifying the poor isolation of key microorganisms in our study, with the “1% culturability paradigm”, which is commonly known as only 1% of microbes are culturable, an application of a wider set of culturing efforts should be done instead, as stated by Martiny^89^. Thus, our results might be an indication that the culture media chosen for this study (MA, PCA, BH), were not adequate in recovering the most relevant genera from the biofilms and should be optimized in the future, by for example including the addition of plastic polymers into a nutrient-poor media.

Furthermore, as Erni-Cassola et al.^24^ suggested in their work, the isolation of potentially interesting taxa could pass through sampling plastic-associated biofilms at earlier stages of surface colonization. In our study, some strains belonging to genera linked to plastic degradation were recover from the one-month-old biofilms, namely some mention in other studies, such as *Bacillus*, *Erythrobacter*, *Exiguobacterium*, *Kocuria*, *Rhodococcus* and *Streptomyces*^37,39,40,42,90^. A marine species of *Pseudomonas* has also been implicated in the degradation of HDPE^91^. So, the potential of each isolated strain obtained in this work, to biodegrade plastic polymers should be accessed in future laboratory experiments.

Overall, marine litter from fisheries is a worrying environmental problem that requires urgent action. Large-scale removal of plastic litter from the ocean, including largely ADLFG, should follow retrieving protocols to avoid additional environmental issues. Therefore, it will be important to promote the implementation of strategies for preventing and reducing marine litter from fisheries, by promoting better practices for waste management^7^ and fishing gear tagging. These strategies will also help mitigate microplastic pollution resulting from the loss and degradation of plastic fishing gear. In addition, this work supports the hypothesis that plastic fishing nets may act as carriers for pathogenic bacteria, potentially transporting harmful microorganisms, which can pose health risks to human populations, either through the consumption of contaminated water or exposure during economic activities carried out in the affected ecosystems. Research on biofilm communities attached to plastic marine litter must also be continued, to enable the isolation of novel microorganisms and the discovery of enzymes capable of breaking down plastics, discovered, thus filling the gap on the current knowledge in plastics degradation.

## Conclusions

Our study represents the first long-term in situ characterization of the microbial community’s succession in plastic fishing nets, inside a marina. Moreover, the comparison with the surrounding seawater communities and isolation of microorganisms from the net biofilms was also achieved. Biofouling of microbial communities and other organisms occurred early on to the fishing nets, where the Thin Nylon net bared a higher biofouling of macroorganisms, such as macroalgae and bivalves, when compared to the other nets, Braided PE or Braided Nylon. Community changes seen on net biofilms were independent of the polymer type or net characteristics. Instead, a temporal succession was observed, which could have been influenced by seasonal changes, biofilm maturation or environmental factors such as nutrient concentrations and biotic interactions with other organisms from the site. Furthermore, communities on biofilms were distinct from the free-living microbial communities in seawater, which were influenced by seasons. In this study, there was no meaningful presence of potentially pathogenic taxa in the net biofilms, nor its release to seawater, with an overall low abundance of these genera, combined: less than 1.5% in total of the net communities. We were also able to identify, in the net biofilms, taxa with hydrocarbon and plastic-degradation potential throughout time, especially after one - month and one-year of incubation. Despite not being able to recover bacteria from the most abundant genera in the one-month-old biofilms, promising bacterial strains, belonging to genera previously reported for hydrocarbon and/or plastic degradation were still isolated. Future work can now explore if the obtained bacterial isolates have plastic polymer degradation capacity. More studies looking into the microbial communities developing in other types of fishing gear, while surveying earlier stages of biofilm succession, could be addressed ahead, to fully comprehend the impacts that ALDFG can pose in marine environments as well as discover the biotechnological potential therein.

## Electronic supplementary material

Supplementary Material 1

## Data Availability

Data is available in ZENODO repository under the DOI 10.5281/zenodo.15585631.
