# Supplementary material for: Microbial communities associated with plastic fishing nets: diversity, potentially pathogenic and hydrocarbon degrading bacteria
Source: Sci Rep. 2025 Jul 2;15:22877. doi: 10.1038/s41598-025-06033-6 (PMC12216815; doi:10.1038/s41598-025-06033-6)
Supplement: Supplementary file 1 — Supplementary Material 1 [file 41598_2025_6033_MOESM1_ESM.pdf]

Supplementary material

Microbial communities associated with plastic fishing nets: Diversity, potentially pathogenic and hydrocarbon degrading bacteria

Perdigão R., Tavares A. S., Carvalho M. F., Magalhães C., Ramos S., Almeida C. M. R., and Mucha A.P.

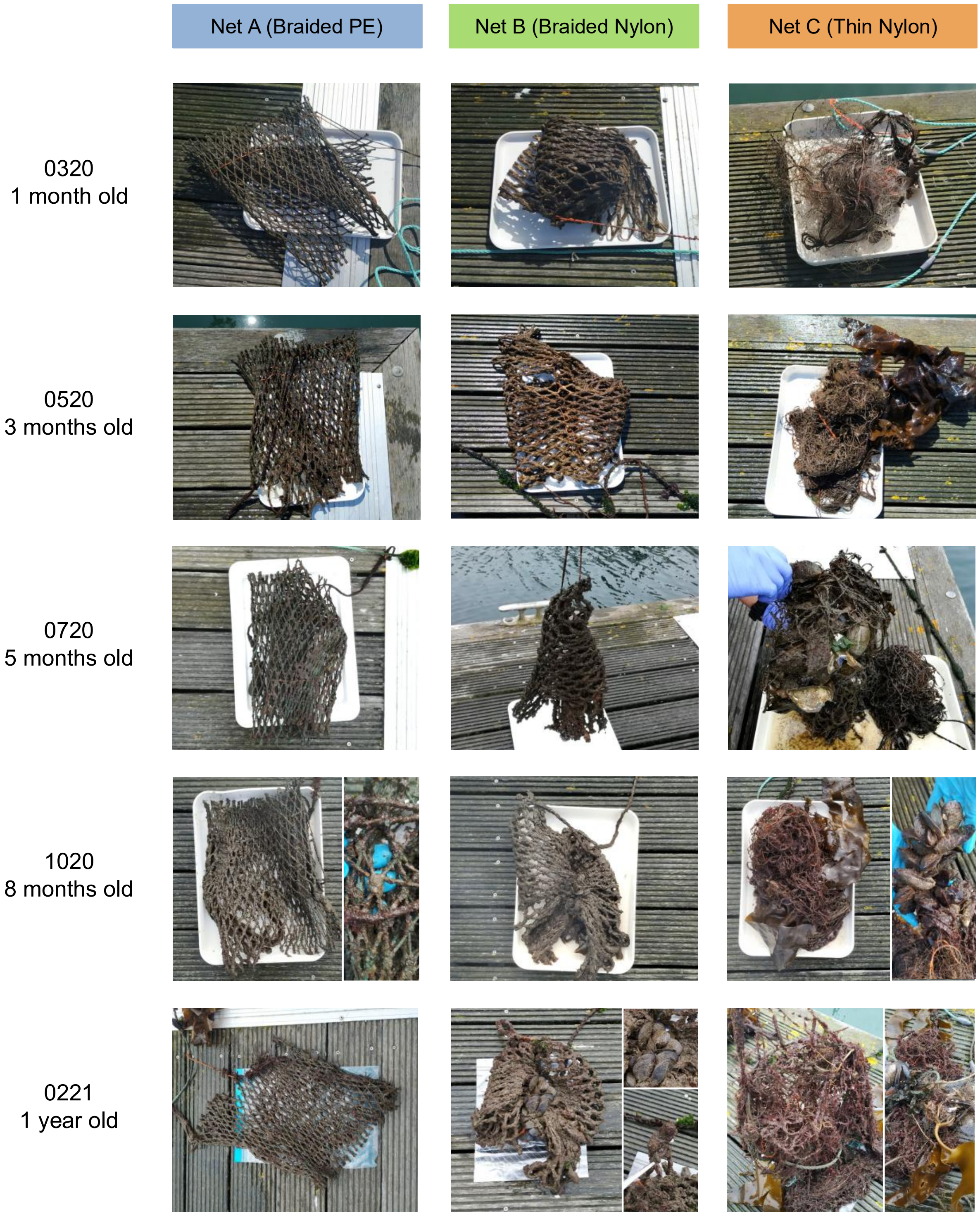

Figure S1. Biofouling succession on fishing nets Braided PE (Net A), Braided Nylon (Net B) and Thin Nylon (Net C), collected from the *in situ* experiment at marina of Leixões, Matosinhos, at the sampling times 0320 (mar_2020), 0520 (may_2020), 0720 (jul_2020), 1020 (oct_2020) and 0221 (fev_2021)..

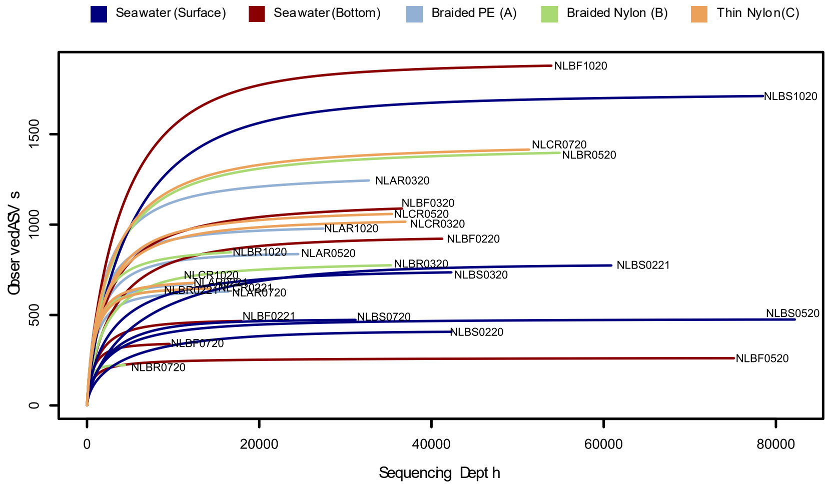

Figure S2. Alpha rarefaction curves of the microbial communities from both net ("NLAR" – Braided PE; "NLBR" - Braided Nylon and "NLCR" - Thin Nylon) and seawater samples at the bottom ("NLBF") and surface ("NLBS"), collected from the in situ experiment of marina of Leixões. Seawater samples were collected at all sampling times (0220 (fev_2020), 0320 (mar_2020), 0520 (may_2020), 0720 (jul_2020), 1020 (oct_2020) and 0221 (fev_2021) while net samples were collected at all times except for 0220 (fev_2020).

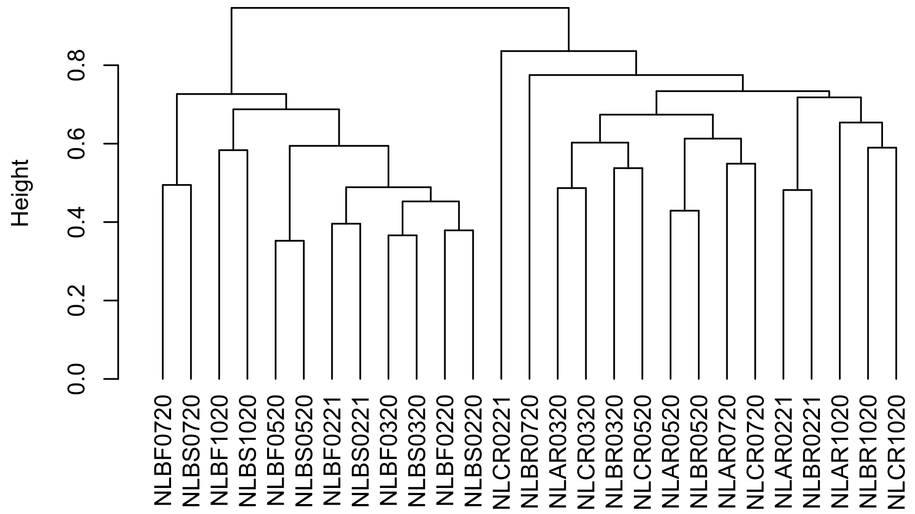

Figure S3. Dendrogram of microbial communities present in both net ("NLAR" – Braided PE; "NLBR" - Braided Nylon and "NLCR" - Thin Nylon) and seawater samples at the bottom ("NLBF") and surface ("NLBS"), collected from the *in situ* experiment of marina of Leixões. Seawater samples were collected at all sampling times (0220 (fev_2020), 0320 (mar_2020), 0520 (may_2020), 0720 (jul_2020), 1020 (oct_2020) and 0221 (fev_2021) while net samples were collected at all times except for 0220 (fev_2020).. Dendrogram done with Hellinger transformed data.

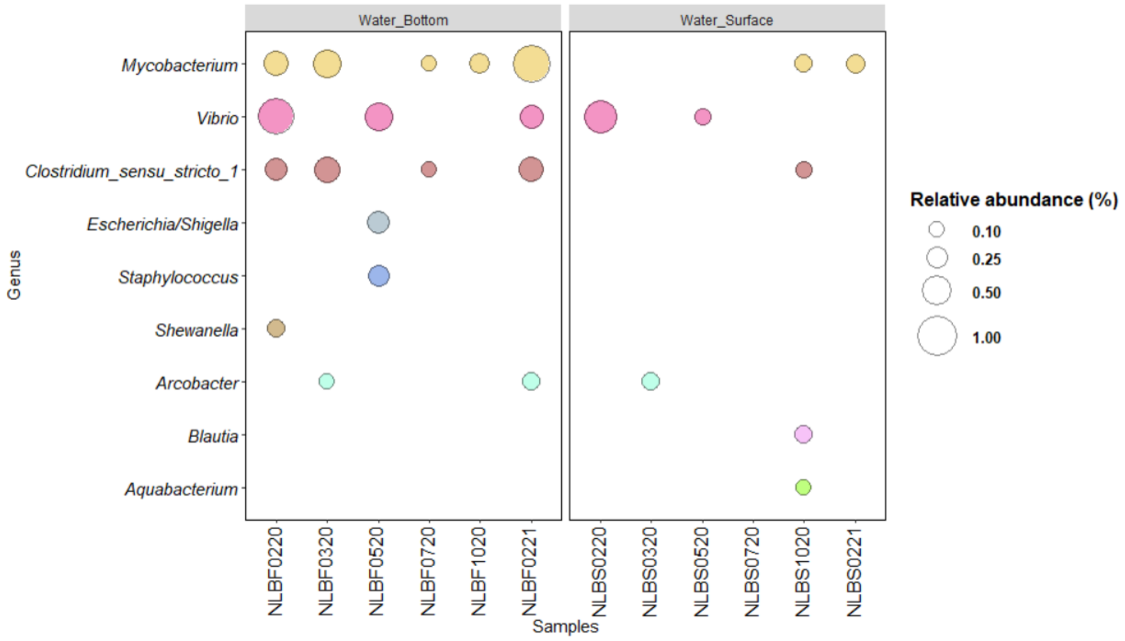

Figure S4. Bubble plot showing the relative abundance (%) of potentially pathogenic groups present in the communities of seawater samples above 0.1% in abundance, collected from the *in situ* experiment at marina of Leixões, at the bottom ("Water_Bottom") and surface ("Water_Surface") and at the sampling times 0220 (fev_2020), 0320 (mar_2020), 0520 (may_2020), 0720 (jul_2020), 1020 (oct_2020) and 0221 (fev_2021).

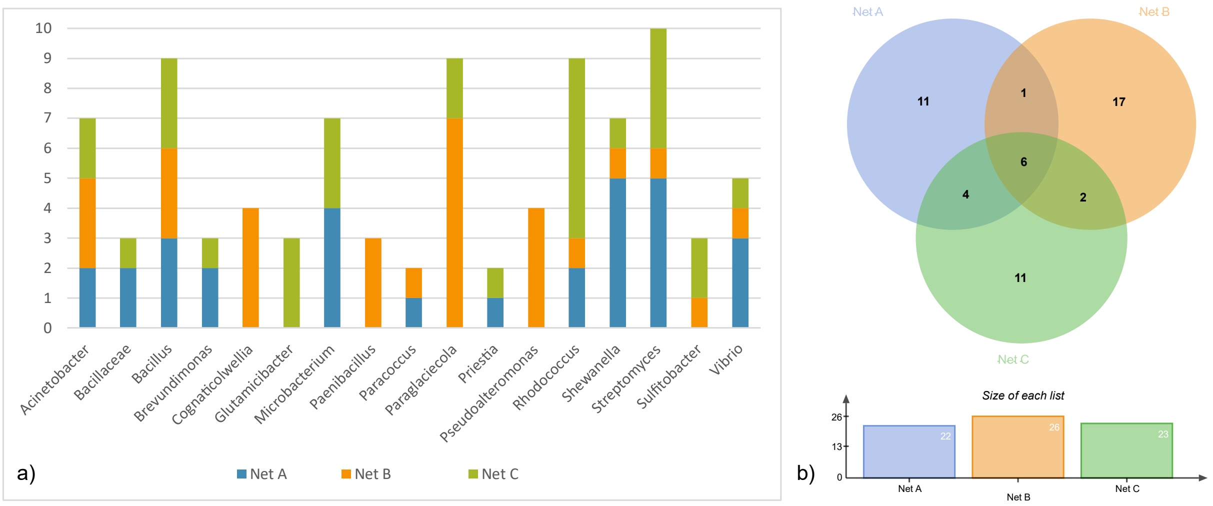

Figure S5. Bacterial genera isolated from the one-month-old biofilm of Braided PE (Net A), Braided Nylon (Net B) and Thin Nylon (Net C) nets, collected from the *in situ* experiment at marina of Leixões: a) Number of bacterial strains isolated more than once, and b) Venn Diagram showing the overall distribution of bacterial genera, in the 3 nets.

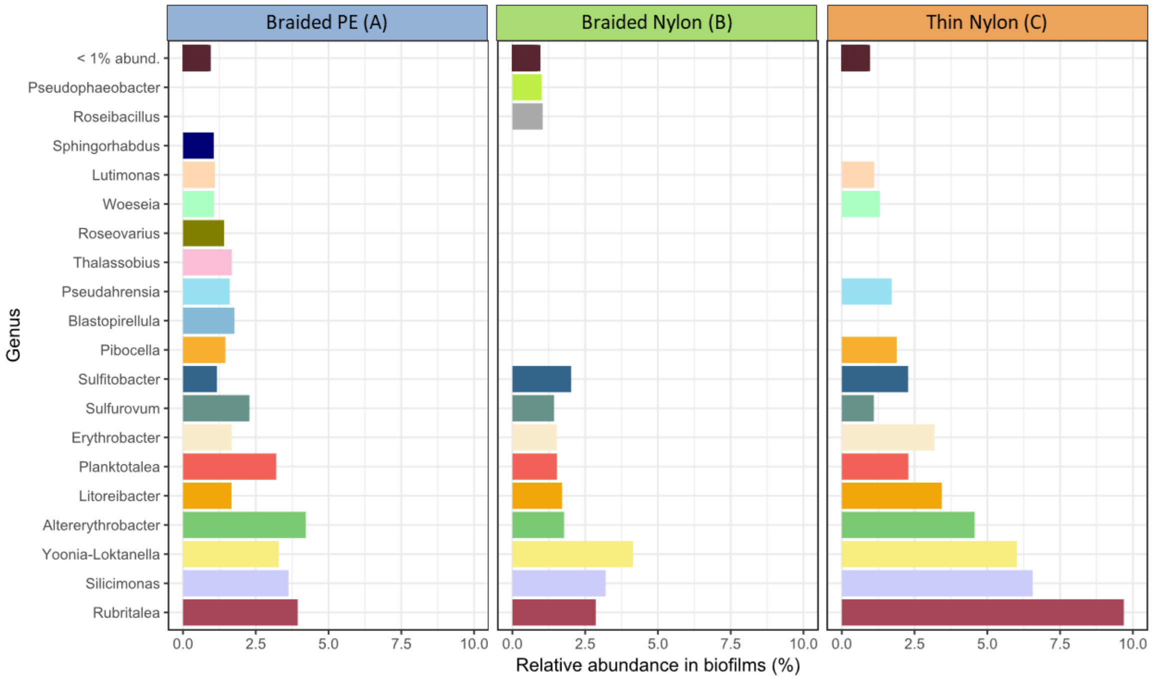

Figure S6. Taxonomic structure until the genus level, of one-month-old biofilm communities present in Braided PE (Net A), Braided Nylon (Net B) and Thin Nylon (Net C) at 0320 above 1% in relative abundance.

Table S1. Phylogenetic identification of bacterial strains isolated one month after the *in situ* experiments from the biofilms of the net A (Braided Polyethylene; NLAR), net B (Braided Nylon; NLBR) and net C (Thin Nylon; NLCR). The bacterial strains were isolated in Marine Agar (MA), Plate Count Agar (PCA) or Bushnell-Haas (BH) solid culture media.

**Isolates**	**Culture media**	**Closest Identification**	**Sequence length**	**% similarity***	**Class**	**Accession number**
NLAR_1	MA	*Shewanella sp.*	1408	99.72	Gammaproteobacteria	OR030648
NLAR_2	MA	*Alkalimarinus sediminis*	1404	98.93	Gammaproteobacteria	OR030560
NLAR_3	MA	*Joostella atrarenae*	1383	98.92	Flavobacteriia	OR030592
NLAR_4	MA	*Vibrio sp.*	1360	100	Gammaproteobacteria	OR030667
NLAR_5	MA	*Vibrio* sp.	1387	99.93	Gammaproteobacteria	OR030670
NLAR_6	MA	*Streptomyces sp.*	1351	99.78	Actinomycetia	OR030658
NLAR_7	MA	*Shewanella sp.*	1362	99.93	Gammaproteobacteria	OR030650
NLAR_9	MA	*Bacillus sp.*	1419	100	Bacilli	OR030566
NLAR_11	MA	*Paracoccaceae* family	1381	99.09	Alphaproteobacteria	OR030631
NLAR_12	MA	*Agarivorans* sp.	1407	99.50	Gammaproteobacteria	OR030558
NLAR_13	MA	*Shewanella sp.*	1391	99.86	Gammaproteobacteria	OR030649
NLAR_15	MA	*Rhodococcus sp.*	1381	100	Actinomycetia	OR030635
NLAR_16	MA	*Microbacterium* sp.	1389	100	Actinomycetia	OR030598
NLAR_18	MA	*Shewanella* sp.	1384	99.93	Gammaproteobacteria	OR030646
NLAR_19	MA	*Kocuria sp.*	1392	99.86	Actinobacteria	OR030593
NLAR_20	MA	*Marinicella sp.*	1410	98.23	Gammaproteobacteria	OR030594
NLAR_22	PCA	Bacillaceae family	1403	99.79	Bacilli	OR030561
NLAR_23	PCA	*Acinetobacter* sp.	1401	99.86	Gammaproteobacteria	OR030552
NLAR_25	PCA	*Shinella* sp.	1352	99.56	Alphaproteobacteria	OR030653
NLAR_26	PCA	Bacillaceae family	1401	99.79	Bacilli	OR030562
NLAR_27	PCA	*Bacillus sp.*	1418	100	Bacilli	OR030564
NLAR_29	PCA	*Bacillus* sp.	1417	100	Bacilli	OR030565

NLAR_30	PCA	*Exiguobacterium oxidotolerans*	1428	99.86	Bacilli	OR030586
NLAR_31	PCA	*Microbacterium sp.*	1391	99.21	Actinomycetia	OR030597
NLAR_32	PCA	*Shewanella* sp.	1380	98.62	Gammaproteobacteria	OR030652
NLAR_33	PCA	*Rhizobium sp.*	1350	99.11	Alphaproteobacteria	OR030633
NLAR_34	PCA	*Priestia sp.*	1422	99.93	Bacilli	OR030621
NLAR_35	PCA	*Microbacterium sp.*	1373	99.56	Actinomycetia	OR030601
NLAR_36	PCA	*Microbacterium sp.*	1392	99.43	Actinomycetia	OR030602
NLAR_37	PCA	*Rhodococcus sp.*	1343	98.66	Actinomycetia	OR030636
NLAR_39	PCA	*Corynebacterium sp.*	1366	99.78	Actinomycetia	OR030582
NLAR_40	PCA	*Brevundimonas sp.*	1329	99.85	Alphaproteobacteria	OR030573
NLAR_41	PCA	*Brevundimonas sp.*	1326	99.7	Alphaproteobacteria	OR030574
NLAR_42	BH	*Acinetobacter sp.*	1369	100	Gammaproteobacteria	OR030554
NLAR_43	BH	*Vibrio sp.*	1429	99.79	Gammaproteobacteria	OR030668
NLAR_44	BH	*Streptomyces sp.*	1373	99.64	Actinomycetia	OR030655
NLAR_46	BH	*Streptomyces sp.*	1389	99.93	Actinomycetia	OR030656
NLAR_47	BH	*Streptomyces* sp.	1400	99.64	Gammaproteobacteria	OR030628
NLAR_48	BH	*Paracoccus sp.*	1329	98.57	Alphaproteobacteria	OR030610
NLAR_49	BH	*Rhodobacteraceae family*	1347	99.93	Alphaproteobacteria	OR030634
NLAR_51	BH	*Streptomyces sp.*	1343	100	Actinomycetia	OR030657
NLBR_1	MA	*Paraglaciecola sp.*	1372	99.64	Gammaproteobacteria	OR030612
NLBR_1.2	MA	*Paraglaciecola sp.*	1346	99.85	Gammaproteobacteria	OR030588
NLBR_2	MA	*Marinomonas profundimaris*	1402	99.29	Gammaproteobacteria	OR030595
NLBR_3	MA	*Shewanella* sp.	1381	99.93	Gammaproteobacteria	OR030647
NLBR_5	MA	*Paraglaciecola sp.*	1377	99.56	Gammaproteobacteria	OR030617
NLBR_6	MA	*Pseudoalteromonas sp.*	1398	100	Gammaproteobacteria	OR030625
NLBR_7	MA	*Cognaticolwellia aestuarii*	1411	99.72	Gammaproteobacteria	OR030580
NLBR_8	MA	*Flavobacteriaceae family*	1346	98.37	Flavobacteriia	OR030587
NLBR_9	MA	*Pseudoalteromonas sp.*	1401	99.93	Gammaproteobacteria	OR030626
NLBR_10	MA	*Paraglaciecola sp.*	1394	99.86	Gammaproteobacteria	OR030613
NLBR_11	MA	*Psychrobacillus* sp.	1420	99.58	Bacilli	OR030632
NLBR_12	MA	*Pseudoalteromonas sp.*	1395	99.64	Gammaproteobacteria	OR030623
NLBR_13	MA	*Cognaticolwellia aestuarii*	1395	99.43	Gammaproteobacteria	OR030578
NLBR_14	MA	*Cognaticolwellia aestuarii*	1357	99.71	Gammaproteobacteria	OR030581
NLBR_15	MA	*Microbacteriaceae family*	1393	99.78	Actinomycetia	OR030596
NLBR_17	MA	*Vibrio sp.*	1404	99.86	Gammaproteobacteria	OR030669
NLBR_18	MA	*Cognaticolwellia aestuarii*	1387	99.78	Gammaproteobacteria	OR030579
NLBR_19	MA	*Sulfitobacter sp.*	1331	99.85	Alphaproteobacteria	OR030665
NLBR_20	MA	*Pseudophaeobacter arcticus*	1324	99.77	Alphaproteobacteria	OR030630
NLBR_22	PCA	*Bacillus sp.*	1415	100	Bacilli	OR030567
NLBR_23	PCA	*Paenibacillus lautus*	1425	99.37	Bacilli	OR030607
NLBR_25	PCA	*Pseudoclavibacter sp.*	1385	99.86	Actinomycetia	OR030627
NLBR_26	PCA	*Bacillus sp.*	1412	100	Bacilli	OR030568
NLBR_27	PCA	*Pseudorhoccus sp.*	1332	99.77	Alphaproteobacteria	OR030611
NLBR_28	PCA	*Paenibacillus sp.*	1420	99.85	Bacilli	OR030609
NLBR_29	PCA	*Acinetobacter* sp.	1404	100	Gammaproteobacteria	OR030551

NLBR_30	PCA	*Streptomyces sp.*	1389	100	Actinomycetia	OR030659
NLBR_32	PCA	*Staphylococcus sp.*	1417	100	Bacilli	OR030654
NLBR_33	PCA	*Acinetobacter sp.*	1368	100	Gammaproteobacteria	OR030555
NLBR_34	PCA	*Salinibacterium amurskyense*	1384	99.93	Actinomycetia	OR030644
NLBR_35	PCA	*Micrococcus sp.*	1376	99.85	Actinomycetia	OR030604
NLBR_36	PCA	*Weeksellaceae* family	1383	99.93	Flavobacteriia	OR030577
NLBR_38	PCA	*Paenibacillus lautus*	1387	99.57	Bacilli	OR030608
NLBR_39	PCA	*Bacillus sp.*	1421	99.86	Bacilli	OR030569
NLBR_40	BH	*Paraglaciecola sp.*	1399	99.43	Gammaproteobacteria	OR030614
NLBR_41	BH	*Acinetobacter lwoffii*	1400	99.57	Gammaproteobacteria	OR030553
NLBR_42	BH	*Pseudoalteromonas sp.*	1402	99.93	Gammaproteobacteria	OR030624
NLBR_43	BH	*Paraglaciecola sp.*	1398	99.71	Gammaproteobacteria	OR030615
NLBR_45	BH	*Paraglaciecola sp.*	1384	99.71	Gammaproteobacteria	OR030616
NLBR_47	BH	*Enterobacteriaceae family*	1387	99.86	Gammaproteobacteria	OR030584
NLBR_48	BH	*Rhodococcus sp.*	1370	99.71	Actinomycetia	OR030637
NLCR_1	PCA	*Rhodococcus sp.*	1350	99.11	Actinomycetia	OR030638
NLCR_2	PCA	*Rhodococcus sp.*	1358	99.93	Actinomycetia	OR030639
NLCR_3	PCA	*Sanguibacter sp.*	1398	99.86	Actinomycetia	OR030645
NLCR_4	PCA	*Acinetobacter sp.*	1389	99.93	Gammaproteobacteria	OR030556
NLCR_5	PCA	*Microbacterium* sp.	1393	100	Actinomycetia	OR030600
NLCR_6	PCA	*Rhodococcus sp.*	1368	99.49	Actinomycetia	OR030642
NLCR_7	PCA	*Microbacterium sp.*	1373	99.85	Actinomycetia	OR030603
NLCR_8	PCA	*Rhodococcus sp.*	1358	99.78	Actinomycetia	OR030643
NLCR_9	PCA	*Pseudomonas sp.*	1391	99.86	Gammaproteobacteria	OR030629
NLCR_12	PCA	*Yersiniaceae family*	1406	99.57	Gammaproteobacteria	OR030672
NLCR_13	PCA	*Oerskovia sp.*	1388	100	Actinomycetia	OR030606
NLCR_16	PCA	*Microbacterium* sp.	1393	99.78	Actinomycetia	OR030599
NLCR_17	PCA	*Bacillus sp.*	1401	99.86	Bacilli	OR030570
NLCR_18	PCA	*Brevundimonas sp.*	1334	99.78	Alphaproteobacteria	OR030575
NLCR_21	PCA	*Glutamicibacter sp.*	1392	99	Actinomycetia	OR030590
NLCR_22	PCA	*Priestia sp.*	1421	99.72	Bacilli	OR030622
NLCR_23	PCA	*Peribacillus sp.*	1424	99.93	Bacilli	OR030620
NLCR_27	PCA	*Bacillus sp.*	1423	99.86	Bacilli	OR030571
NLCR_28	PCA	*Glutamicibacter sp.*	1377	99.93	Actinomycetia	OR030591
NLCR_29	PCA	*Bacillus sp.*	1421	99.86	Bacilli	OR030572
NLCR_31	MA	*Agrococcus sp.*	1372	99.85	Actinobacteria	OR030559
NLCR_32	MA	*Cellulophaga tyrosinoxydans*	1383	99.13	Flavobacteriia	OR030576
NLCR_33	MA	*Erythrobacteraceae* family	1350	98.89	Alphaproteobacteria	OR030585
NLCR_35	MA	*Streptomyces sp.*	1370	99.64	Actinomycetia	OR030660
NLCR_36	MA	*Sulfitobacter sp.*	1322	98.67	Alphaproteobacteria	OR030666
NLCR_41	MA	*Vibrio* sp.	1417	100	Gammaproteobacteria	OR030671
NLCR_42	MA	*Shewanella sp.*	1405	99.93	Gammaproteobacteria	OR030651
NLCR_44	MA	*Rhodococcus sp.*	1383	100	Actinomycetia	OR030640
NLCR_45	MA	*Sulfitobacter geojensis*	1319	99.85	Alphaproteobacteria	OR030664
NLCR_47	MA	Bacillaceae family	1417	99.79	Bacilli	OR030563
NLCR_48	MA	*Nocardiaceae* family	1387	99.57	Actinomycetia	OR030605
NLCR_49	MA	*Paraglaciecola sp.*	1395	99.79	Gammaproteobacteria	OR030618
NLCR_51	BH	*Streptomyces sp.*	1370	99.93	Actinomycetia	OR030661

NLCR_52	BH	*Acinetobacter sp.*	1401	99.93	Gammaproteobacteria	OR030557
NLCR_53	BH	*Rhodococcus sp.*	1382	99.93	Actinomycetia	OR030641
NLCR_54	BH	*Dietzia sp.*	1383	99.86	Actinomycetia	OR030583
NLCR_55	BH	*Glutamicibacter protophormiae*	1375	99.93	Actinomycetia	OR030589
NLCR_57	BH	*Streptomyces sp.*	1392	100	Actinomycetia	OR030662
NLCR_58	BH	*Paraglaciecola sp.*	1390	99.86	Gammaproteobacteria	OR030619
NLCR_59	BH	*Streptomyces sp.*	1393	100	Actinomycetia	OR030663

Table S2. Environmental parameters from seawater samples collected in site A (Braided PE), B (Braided Nylon) or C (Thin Nylon), from the *in situ* experiment at marina of Leixões. Values represent average ± SD.

Sampling time	Sampling site	Temperature (ºC)	Salinity (psu)	Dissolved O_2 (mg. L^{-1})	pH	Turbidity (FNU)
0220	A	13.90 ± 0.08	28.44 ± 2.41	6.53 ± 0.13	7.95 ± 0.01	0.95 ± 0.79
	B	13.94 ± 0.08	28.22 ± 2.70	6.59 ± 0.14	8.04 ± 0.02	1.12 ± 1.04
	C	13.91 ± 0.05	27.85 ± 4.20	7.21 ± 0.42	8.07 ± 0.02	2.18 ± 2.77
0320	A	14.02 ± 0.04	31.27 ± 0.89	6.78 ± 0.06	8.35 ± 0.00	4.65 ± 2.40
	B	14.01 ± 0.03	30.73 ± 0.52	6.77 ± 0.04	8.41 ± 0.00	3.14 ± 1.30
	C	14.03 ± 0.05	31.34 ± 0.86	6.68 ± 0.07	8.42 ± 0.01	4.51 ± 2.18
0520	A	17.80 ± 0.15	32.65 ± 1.24	**	8.69 ± 0.03	0.69 ± 0.93
	B	17.98 ± 0.24	33.10 ± 1.83	**	8.75 ± 0.03	2.43 ± 2.83
	C	17.86 ± 0.12	32.03 ± 1.30	**	8.82 ± 0.02	0.47 ± 0.17
0720	A	17.95 ± 0.59	35.48 ± 0.35	**	8.42 ± 0.00	0.34 ± 0.18
	B	17.74 ± 0.60	35.57 ± 0.39	**	8.44 ± 0.00	0.60 ± 0.37
	C	17.78 ± 0.61	35.53 ± 0.43	**	8.45 ± 0.00	0.51 ± 0.28
1020	A	15.33 ± 0.03	33.39 ± 0.79	6.28*	**	7.23 ± 0.93
	B	15.39 ± 0.03	31.72 ± 0.58	7.42*	**	4.81 ± 1.05
	C	15.39 ± 0.03	31.78 ± 0.60	7.39*	**	4.77 ± 0.99
0221	A	13.53 ± 0.21	28.28 ± 2.79	**	7.69 ± 0.00	1.78 ± 0.69
	B	13.58 ± 0.13	28.75 ± 1.82	**	7.74 ± 0.00	1.93 ± 0.70
	C	13.53 ± 0.20	28.49 ± 2.23	**	7.78 ± 0.01	1.54 ± 0.34

*Measurements made with another multiparameter probe. Values represent a single read.
**multiparameter probe sensor malfunction.

Table S3. Inorganic nutrients content (Nitrite (NO_3^-), Nitrate (NO_3^-), Ammonium ($NH_4^+ + NH_3$), Phosphate (PO_4^{3-})), Chlorophyll a concentration, Total Particulate Matter (TPM) and Particulate Organic Matter (POM) of surface (S) and bottom (F) seawater samples collected in site A (Braided PE), B (Braided Nylon) or C (Thin Nylon), from the *in situ* experiment at marina of Leixões. Values represent average ± SD in nutrients, and concentration in Chl a, TPM, POM.

Time	Site	NO_2^- (µM. L^{-1})	NO_3^- (µM. L^{-1})	$NH_4^+ + NH_3$ (µM. L^{-1})	PO_4^{3-} (µM. L^{-1})	Chl a (µg. L^{-1})	TPM (mg. L^{-1})	POM (mg. L^{-1})
0220	AS	3.01 ± 0.06	73.68 ± 0.70	25.26 ± 0.18	2.27 ± 0.02	0.31	0.03	0.0058
	AF	1.34 ± 0.00	46.32 ± 0.62	12.50 ± 0.10	1.58 ± 0.08	1.79	0.04	0.007
	BS	2.40 ± 0.04	72.50 ± 2.69	21.81 ± 0.20	1.58 ± 0.04	0.29	0.03	0.0064
	BF	0.96 ± 0.02	34.78 ± 0.18	9.86 ± 0.06	1.21 ± 0.02	2.13	0.04	0.0072
	CS	3.05 ± 0.06	120.13 ± 1.65	25.76 ± 0.12	2.62 ± 0.02	0.41	0.03	0.0052
	CF	1.09 ± 0.03	43.61 ± 0.12	10.45 ± 0.18	1.33 ± 0.02	2.82	0.04	0.0064
0320	AS	1.62 ± 0.05	27.48 ± 0.16	11.89 ± 0.10	1.84 ± 0.23	0.51	0.04	0.0056
	AF	0.74 ± 0.08	26.74 ± 0.41	6.60 ± 0.09	1.31 ± 0.18	1.01	0.11	0.0182
	BS	1.35 ± 0.09	48.45 ± 1.45	10.47 ± 0.10	1.61 ± 0.04	0.48	0.04	0.0052

	BF	0.80 ± 0.00	31.26 ± 0.38	7.57 ± 0.31	1.11 ± 0.09	0.23	0.06	0.0078
	CS	1.87 ± 0.26	62.82 ± 0.36	11.79 ± 0.13	1.84 ± 0.14	0.51	0.05	0.0056
	CF	1.27 ± 0.02	33.82 ± 0.12	10.17 ± 0.17	1.78 ± 0.02	0.25	0.04	0.0058
0520	AS	1.62 ± 0.06	36.61 ± 0.79	23.55 ± 0.20	3.17 ± 0.07	0.96	0.03	0.0058
	AF	0.70 ± 0.09	16.91 ± 0.18	8.77 ± 0.19	1.45 ± 0.10	1.36	0.04	0.009
	BS	1.34 ± 0.10	48.98 ± 2.16	23.24 ± 0.25	2.54 ± 0.06	0.77	0.04	0.0084
	BF	0.77 ± 0.00	22.26 ± 0.27	6.56 ± 0.30	1.42 ± 0.02	0.57	0.05	0.0084
	CS	1.88 ± 0.28	58.14 ± 1.68	24.80 ± 0.14	3.14 ± 0.02	1.11	0.04	0.0068
	CF	1.26 ± 0.02	11.04 ± 0.41	8.90 ± 0.07	1.60 ± 0.06	1.60	0.05	0.0104
0720	AS	0.61 ± 0.03	14.23 ± 0.10	3.54 ± 0.23	1.48 ± 0.04	1.57	0.07	0.00933
	AF	0.63 ± 0.10	9.53 ± 0.08	2.04 ± 0.17	1.37 ± 0.31	7.17	0.05	0.0096
	BS	0.54 ± 0.04	14.93 ± 0.02	2.59 ± 0.26	1.31 ± 0.06	1.54	0.04	0.0074
	BF	0.73 ± 0.03	8.10 ± 0.50	2.17 ± 0.17	1.18 ± 0.02	2.62	0.04	0.0088
	CS	0.37 ± 0.05	14.64 ± 1.10	2.38 ± 0.10	1.07 ± 0.04	1.99	0.04	0.0088
	CF	0.66 ± 0.12	9.24 ± 0.51	1.91 ± 0.33	0.94 ± 0.10	7.42	0.04	0.0096
1020	AS	1.40 ± 0.08	33.07 ± 0.37	10.17 ± 1.31	1.31 ± 0.03	2.47	0.06	0.01084
	AF	0.76 ± 0.00	12.04 ± 0.60	6.71 ± 0.25	0.87 ± 0.02	16.82	0.15	0.02864
	BS	1.66 ± 0.06	32.69 ± 1.82	11.12 ± 1.66	1.38 ± 0.11	2.39	0.04	0.00896
	BF	1.01 ± 0.42	10.59 ± 0.91	6.38 ± 0.26	0.66 ± 0.05	1.97	0.06	0.01198
	CS	1.37 ± 0.20	23.32 ± 1.30	9.45 ± 0.99	1.22 ± 0.02	2.52	0.05	0.01204
	CF	0.87 ± 0.07	18.95 ± 0.78	7.64 ± 0.25	1.17 ± 0.02	4.12	0.06	0.01202
0221	AS	1.56 ± 0.14	134.40 ± 11.10	11.45 ± 0.57	1.99 ± 0.03	0.36	0.03	0.00716
	AF	1.00 ± 0.05	43.85 ± 2.81	5.79 ± 0.08	1.19 ± 0.09	1.82	0.04	0.00946
	BS	1.40 ± 0.03	76.45 ± 1.81	13.21 ± 3.87	1.31 ± 0.00	0.40	0.03	0.00638
	BF	1.12 ± 0.22	45.76 ± 4.02	6.31 ± 0.14	1.24 ± 0.16	0.51	0.04	0.00818
	CS	1.28 ± 0.23	91.91 ± 6.89	10.19 ± 0.12	1.71 ± 0.35	0.19	0.03	0.00678
	CF	0.74 ± 0.05	38.87 ± 2.59	4.92 ± 0.18	1.34 ± 0.19	4.71	0.08	0.01424

Table S4. Number of raw sequences (input), obtained after NGS and number of sequences obtained throughout the preprocessing steps of the DADA2 pipeline. Samples of seawater, from the surface (NLBS) and bottom (NLBF), and net biofilms Braided PE (NLAR), Braided Nylon (NLBR) and Thin Nylon (NLCR), collected from the *in situ* experiment at marina of Leixões, Matosinhos. Samples were collected at the beginning, February of 2020 (0220), and in the months of March (0320), May (0520), July (0720), October (1020) and after 1 year of experiment, in February 2021 (0221).

Samples	Input	Filtered	DenoisedF	DenoisedR	Merged	Non-chimeric
NLAR0221	33522	24709	20712	22491	15385	13286
NLAR0320	83421	67231	58410	63124	44580	37523
NLAR0520	68684	43198	39586	41112	33366	28771
NLAR0720	38608	29083	26011	27596	20759	17594
NLAR1020	57911	46118	41642	43900	33112	28057
NLBF0220	93912	73319	65306	69328	52341	42570
NLBF0221	36727	29482	25933	27592	21129	18396
NLBF0320	86462	68250	59301	63549	46416	38885
NLBF0520	126920	92921	92424	92748	86009	84232
NLBF0720	35880	30087	27940	28841	25259	22698
NLBF1020	86174	69251	66742	67571	60966	57490
NLBR0221	29138	22760	18558	20760	13718	11775
NLBR0320	91399	78723	74058	76552	65498	51397

NLBR0520	145325	90915	84505	87287	72116	61486
NLBR0720	15675	9360	8584	8826	7200	6235
NLBR1020	41321	31853	26940	29354	20032	17417
NLBS0220	76570	53429	51667	52292	47514	42907
NLBS0221	94273	74003	72371	72814	67158	61576
NLBS0320	95904	76734	70398	73273	58352	44597
NLBS0520	140135	99377	98132	98854	93690	88076
NLBS0720	66455	55803	55221	55422	53391	50577
NLBS1020	111832	90677	88827	89615	82965	79433
NLCR0221	35809	26257	22703	24335	18052	16389
NLCR0320	93935	61683	56690	58934	48257	41758
NLCR0520	95247	73728	67507	70786	55951	46274
NLCR0720	116910	96018	88811	94085	70586	55241
NLCR1020	30969	24065	20272	22281	15674	13814
